# The Impact of Elevated Soil pH Levels on Cranberry Growth, Physiology, and Metabolites

**DOI:** 10.3390/plants14182833

**Published:** 2025-09-11

**Authors:** Mura Jyostna Devi, Jinyoung Barnaby, Jessica Rohde, Yi Wang, Lorraine Rodriguez-Bonilla, Juan Zalapa, Amaya Atucha, Giverson Mupambi

**Affiliations:** 1 Vegetable Crops Research Unit, US Department of Agriculture (USDA)—Agricultural Research Service (ARS), Madison, WI 53706, USA; 2 Department of Plant and Agroecosystem Sciences, University of Wisconsin, Madison, WI 53706, USA; 3 US Department of Agriculture (USDA)—Agricultural Research Service (ARS), Adaptive Cropping Systems Laboratory, Beltsville Agriculture Research Center, Beltsville, MD 20705, USA; 4 Cranberry Station Facility, University of Massachusetts Cranberry Station, One State Bog Rd., East Wareham, MA 02538, USA

**Keywords:** cranberry, high soil pH, photosynthesis, chlorophyll fluorescence, nutrients, metabolites

## Abstract

pH plays a critical role in regulating nutrient availability and uptake, directly influencing plant growth and productivity. Cranberries grow optimally within a soil pH range of 4.2 to 5.5, but achieving this range remains challenging, even with amendments. This study evaluated the effects of elevated soil pH (7.0 to 7.2) on cranberry cultivation and identified factors contributing to adverse outcomes. Stems, leaves, and fruits were sampled from plants grown in soil with a pH ranging from 4.8 to 7.0. Nutrient composition, fruit size, yield, and anthocyanin content were analyzed. High soil pH levels resulted in significant reductions in fruit size (25–35%) and yield (29–56%). Cranberry plants grown in elevated pH conditions showed a significant (*p* < 0.001) decline in nitrogen, phosphorus, and potassium, and increased calcium and magnesium in soil, stems, leaves, and fruits. Additionally, photosynthesis and chlorophyll fluorescence were significantly reduced (*p* < 0.05 to 0.001). Certain amino acids, carbohydrates, and organic acids increased significantly (*p* < 0.05 to 0.0001) in high pH soils, suggesting a role in stress adaptation. Calcium levels in fruits and shoots were inversely correlated with fruit size and some metabolites. These findings demonstrate that soil pH levels above the optimal range (4.2–5.5) substantially impair cranberry growth and quality by disrupting nutrient balance and photosynthesis. The results highlight the urgent need for improved water and soil management strategies to mitigate high soil pH stress in commercial cranberry production.

## 1. Introduction

The American cranberry (*Vaccinium macrocarpon* L.), a member of the Ericaceae family, is a highly valuable fruit crop due to its significant economic value and abundance of vitamin C, several nutrients, and antioxidants [1]. The United States is the largest producer of cranberries, accounting for 68% of the global total, with Wisconsin as the leading state [2]. Cranberry production has steadily increased since 1990, as year-round consumption has led to a rise in supply. This increase is partially attributable to the rise in processed cranberries, primarily juice and juice blends [3].

Soil is a complex medium that plays a crucial role in plant growth and development. It provides water, nutrients, and anchorage for plants and mediates plant–soil–microbe interactions [4]. It is referred to as the master variable, as it controls numerous chemical and biological processes in the soil system, including soil characteristics, nutrient absorption, soil remediation, enzyme activity, microbes, decomposition, nitrification, and denitrification [5]. The cranberry plant is classified as a calcifuge species, exhibiting optimal growth within a soil pH range of 4.2 to 5.5. However, soil pH levels have been reported to vary across different cranberry-growing regions [6]. The sandy soils in which cranberries are cultivated possess limited buffering capacity, leading to rapid pH fluctuations following the application of elemental sulfur, ammonium-based fertilizers, and other soil amendments [7]. Given that cranberry production systems are highly dependent on water resources, water quality represents an additional factor influencing soil pH. A study by Hanson and Stein (1999) [8] demonstrated that the use of alkaline irrigation water in cranberry fields significantly increased soil pH, thereby affecting nutrient availability [8]. Most micronutrients are more readily available to plants in acidic soils than in neutral to alkaline conditions, which supports plant growth [9]. However, certain micronutrients and non-essential elements may become toxic when present at elevated concentrations. While most macronutrients tend to be more available in alkaline soils, the availability of phosphorus and micronutrients is typically reduced, potentially leading to nutrient imbalances. Such imbalances can negatively impact plant growth and fruit production [10].

At elevated pH levels, the availability of phosphorus and micronutrients such as Fe, Mn, Zn, and Cu declines, while calcium and magnesium often increase, leading to nutrient imbalances—a pattern supported by recent findings in lime- and vermicompost-amended acidic soils [11]. Conversely, at low pH, Al^3+^ toxicity becomes a major constraint, severely inhibiting root growth, disrupting nutrient uptake, and altering membrane integrity and antioxidant responses [12]. Previous studies on blueberries, also of the *Vaccinium* genus, sharing similar demands to soil pH levels as cranberries, have indicated that a soil pH level above 5 negatively impacted both yield and fruit size [13]. Several studies have indicated that an increase in pH levels within the rhizosphere of blueberry plants can result in a disruption of nutrient balance, occurrence of leaf iron deficiency chlorosis, and a reduction in photosynthetic activity, impeding overall growth of the plant [10,14]. Blueberry plants exhibited nutrient deficiency, inhibited growth, and diminished fruit yield and quality when the pH of the soil surpassed 6.0. The reduced yield can be attributed to reduced utilization rate of macro- and micronutrients in high pH soil levels that are not optimal for supporting the nutritional needs and growth of blueberry plants. Further, blueberry flower bud differentiation and flowering phenology were delayed under high soil pH conditions (pH 6.0), reducing fruit quality and increasing fruit titratable acid content [15].

Chlorophyll fluorescence is a widely used indicator of plant stress, with parameters such as Fv/Fo, Fv/Fm, and Y(II) providing insight into photosynthetic performance under both dark- and light-adapted conditions [16]. Fv/Fm is particularly sensitive, with reduced values indicating stress, photoinhibition, or downregulation of photosynthesis. Studies in blueberries have shown that high soil pH decreases chlorophyll content and Fv/Fm, likely due to disruption of membrane integrity and photosynthetic function [10]. Elevated malondialdehyde content, reflecting oxidative damage, was also negatively correlated with growth under high pH soils [15].

Plants can modify their physiological functions to adapt to stressful conditions by regulating the concentrations of some metabolites, including proline, betaine, soluble sugars, polyamines, glutathione, ascorbic acid, etc. [17,18]. Metabolites, such as amino acids, carbohydrates, lipids, polyamines, and glycine betaine, are regarded as the most important factors that serve as osmolytes and osmoprotectants against environmental stress in plants, protecting cell membranes [17]. Metabolites such as sugars, amino acids, and organic acids play a significant role in cranberry fruits, as they contribute to the distinctive flavor profile of these fruits. Organic acids play a critical role in determining fruit quality attributes such as maturity and flavor [19]. Metabolic components may play a role in high pH stress responses; however, knowledge of high pH soil-related metabolomic components is limited.

Although extensive work has been conducted on blueberries and other Ericaceae species under different soil pH conditions, studies on cranberries are limited [8,11,15]. For example, Finn (1990) [20] demonstrated that cranberries grown in solution culture at higher pH (6.5 compared to 4.5) developed altered root branching, potentially affecting nutrient uptake. Similarly, Szwonek et al. (2016) [21] reported cranberry growth and yield performance on mineral soils, confirming the crop’s dependence on low pH conditions. These findings highlight the importance of pH management, yet the specific physiological and metabolic responses of cranberry to elevated pH remain poorly documented. Importantly, cranberry growers in some regions are increasingly encountering the challenge of elevated soil pH, largely due to irrigation water with high calcium content, which has become a persistent constraint on crop performance. Our study was therefore motivated not only by scientific gaps in the literature but also by the need to address this real-world problem faced by farmers. Accordingly, the present study addresses this gap by evaluating the effects of elevated soil pH (7.0–7.2) on cranberry growth, nutrient availability and uptake, photosynthesis, and metabolite responses. By linking nutrient profiles with physiological and biochemical markers, we aim to clarify the mechanisms underlying reduced fruit yield and quality under high pH stress and provide insights that may inform management practices for growers.

## 2. Results

### 2.1. Soil pH, Water pH, and Nutrients

The results indicated that the average soil pH measured at different time intervals (>3 times per year) over three years was significantly higher (*p* < 0.01) in four high cranberry beds (7.0 to 7.2) than in the control (4.8 ± 0.04) (Table 1). Also, the irrigation water samples supplied to high-pH soil beds exhibited significantly elevated levels of pH (Table 1) and total hardness, alkalinity, and conductivity in comparison to the control group (Table S1). The control cranberry soils exhibited a higher percentage of organic matter and available phosphorus compared to the four high-soil-pH beds. Nevertheless, the concentrations of total phosphorus and potassium were observed to be comparable in soils with high pH levels of 3 and 4. In contrast, calcium and magnesium levels, and their respective percentages, were significantly elevated (*p* < 0.001) under high soil pH conditions. Micronutrients, including manganese, zinc, copper, and boron, differed significantly between high pH and control soils. The nitrogen content was observed to be comparatively low in high pH1 and high pH2 soils. However, there was no difference in nitrogen levels between high pH3, pH4, and control soils. Like organic matter, carbon levels were found to be significantly greater in control soils than in high pH (Table 1 and Table S2).

### 2.2. Fruit Traits

#### 2.2.1. Fruit Size

**Figure 1:**
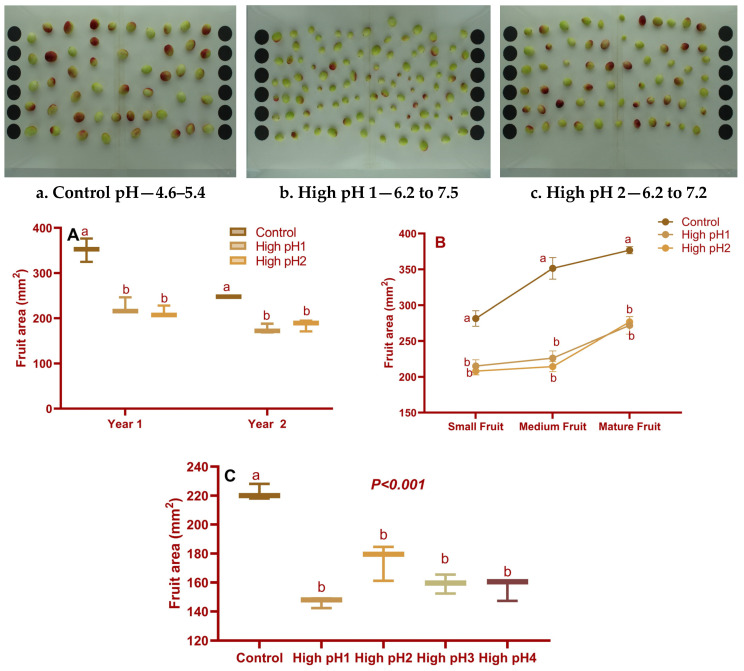
Fruit area of control and high soil pH fruits. Images of harvested fruits from control (**a**), high pH1 (**b**), and high pH2 (**c**) cranberry beds. (**A**): Average fruit area (mm^2^) of mature fruits collected in years 1 and 2; (**B**): Average fruit area (cm^2^) of small, medium, and mature fruits in year 1; (**C**): Average fruit area (cm^2^) of fruits from four high soil pH beds and control beds in year 3. The bars or dots represented with different lowercase alphabets are significantly different according to the Tukey-Kramer model at *p* < 0.05.

The fruit area was reduced in all high-pH soils across the three-year experimental period (Figure 1A–C). (The fruits grown in the control soil pH exhibited larger sizes compared to those cultivated in high soil pH1 and pH2. In the first year, there was a reduction in fruit area ranging from 35 to 39%, while in the second year, it was from 25 to 29%. In the third year of the study, it was observed that high pH1 treatment resulted in the smallest fruit area, measuring 146 cm^2^. This represented a decrease of 34% when compared to the control group (Figure 1C). Furthermore, the dimensions of the fruit, including both length and width, exhibited varying degrees of reduction in all four high soil pH fruits compared to the control (Figure S1A–H).

#### 2.2.2. Fruit Yield and Weight

**Figure 2:**
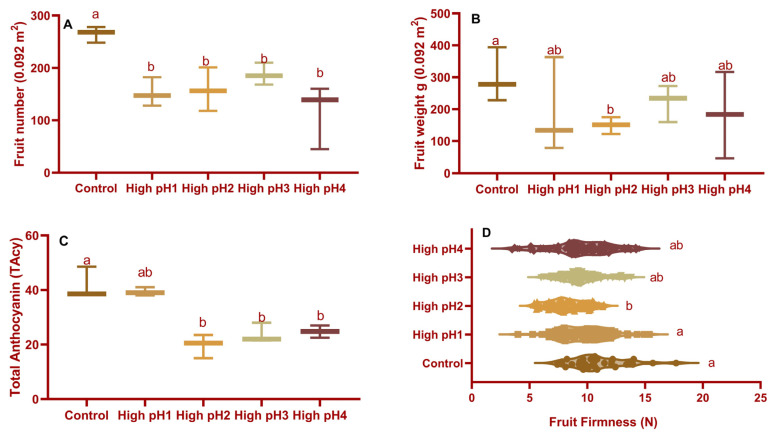
Fruit parameters of fruits collected from four different high soil pH cranberry beds along with an optimum (control) soil pH bed. Average ± S.E. of (**A**) Fruit number (**B**) Fruit weight (**C**) Total anthocyanin content and (**D**) Fruit firmness of the fruits collected at the mature stage in year 3. The bars represented with similar small alphabets are not significantly different based on the Tukey-Kramer model at *p* < 0.05.

The yield, calculated as the number of fruits per 0.09 m^2^, was found to be significantly different (*p* < 0.0001) in all high pH soil beds compared to the control (Figure 2A), with percentage reductions ranging from 29 to 56%. There were no differences in fruit number across high-pH soil beds. Under high soil pH stress, the fruit weight of only high pH2 decreased (Figure 2A,B).

#### 2.2.3. Fruit Color and Firmness

There were significant differences (*p* < 0.05 to <0.001) observed in the fruit’s redness or total anthocyanin content and firmness among the treatments. The percentage of reduction in fruit color in high pH2, 3, and 4 beds ranged from 40% to 52%. However, no change in fruit color was observed in high pH1 treatment fruits (Figure 2C,D).

### 2.3. Gas Exchange Parameters

**Figure 3:**
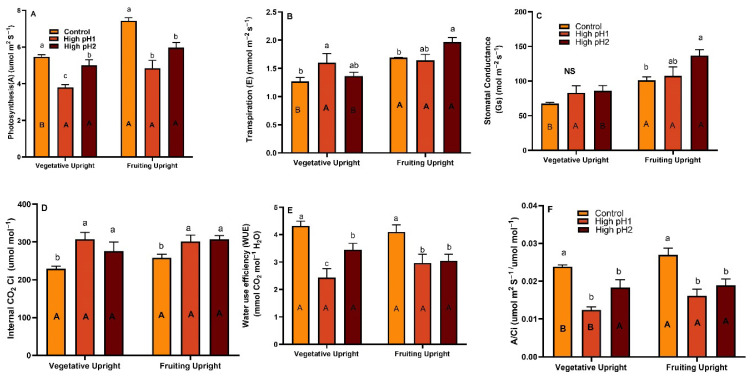
Photosynthesis, transpiration, stomatal conductance, Ci, WUE, and A/Ci in vegetative and fruiting cranberry uprights under optimum and high pH conditions. Average ± S.E. of (**A**) Photosynthesis, (**B**) Transpiration, (**C**) Stomatal conductance, (**D**) Internal Ci, (**E**) Water Use efficiency, and (**F**) A/Ci measured in vegetative and reproductive uprights in year 3 in high pH1, high pH2, and control beds. The bars denoted by similar lowercase letters do not exhibit statistically significant differences across treatments for vegetative and reproductive uprights, and the bars represented by uppercase letters are for the differences across upright types within the treatment, as determined by Tukey’s-Kramer model at *p* < 0.05.

The cranberry plants subjected to high soil pH stress experienced a reduction in photosynthesis during the early growing season and fruit development stage. This inhibition was statistically significant (*p* < 0.01) in both stages compared to the control plants (Figure 3 and Figure S2). The vegetative and fruiting uprights in the high pH1 soil exhibited photosynthesis rates of 3.8 and 4.8 µmol m^2^ S^−1^, respectively, which were lower compared to the control and high pH2 soils. The study observed that the rate of photosynthesis in fruiting upright shoots was higher compared to vegetative upright shoots. This difference was found to be statistically significant under normal conditions (Figure 3A). The transpiration and stomatal conductance significantly increased in fruiting uprights (*p* < 0.05) of high pH soil plants (*p* < 0.01). Nevertheless, the WUE of control plants exhibited a notable increase in vegetative (*p* < 0.0001) and fruiting upright (*p* < 0.001) shoots, with values of 4.3 and 4.1 mmol CO_2_ mol^−1^ H_2_O, respectively. Both high pH1 and pH2 soil treatments showed a decrease in WUE when compared to the control plants (Figure 3E). The concentration of internal CO_2_ (Ci) ranged from 275 to 308 µmol mol^−1^ in cranberry plants with high pH soils, which was higher compared to the control plants. There was no significant difference in the specific leaf area (SLA) between the control and pH treatment groups in vegetative upright, and fruiting uprights were observed.

### 2.4. Chlorophyll Fluorescence

**Figure 4:**
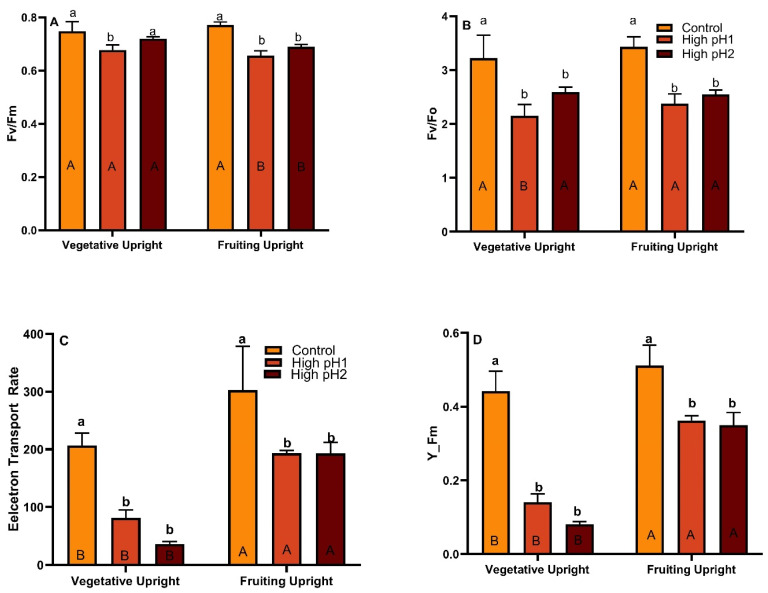
Chlorophyll fluorescence parameters in vegetative and fruiting cranberry uprights under optimum and high pH conditions. Average ± S.E. of (**A**) Fv/Fm (**B**) Fv/Fo (**C**) Electron transport rate and (**D**) YII measured in vegetative and reproductive uprights in year 3 in high pH1, high pH2, and control beds. The bars represented by similar lowercase letters do not exhibit statistically significant differences across treatments for both vegetative and reproductive uprights, and the bars represented by the same uppercase letters do not show significant differences across upright types of the same treatment, as determined by Tukey’s-Kramer model at *p* < 0.05.

There was a significant variation observed in the values of chlorophyll fluorescence among treatments with high and control soil pH. Overall, the values of Fv/Fm and Fv/F0, measured under dark-adapted conditions, showed reduced levels in high-pH plants as compared to the control in both vegetative and reproductive uprights (*p* < 0.05 to *p* < 0.001) (Figure 4A,B). Nevertheless, notable variation was detected in high soil pH plants between the two categories of uprights. The light-adapted stress measurements Y(II) and ETR of plants cultivated under high soil pH conditions demonstrated a significant decrease (*p* < 0.0001) in both parameters when compared to control plants (Figure 4C,D).

### 2.5. Stem, Leaf, and Fruit Nutrients

#### 2.5.1. Leaves, Stems, and Shoots Macro and Micronutrients

**Figure 5:**
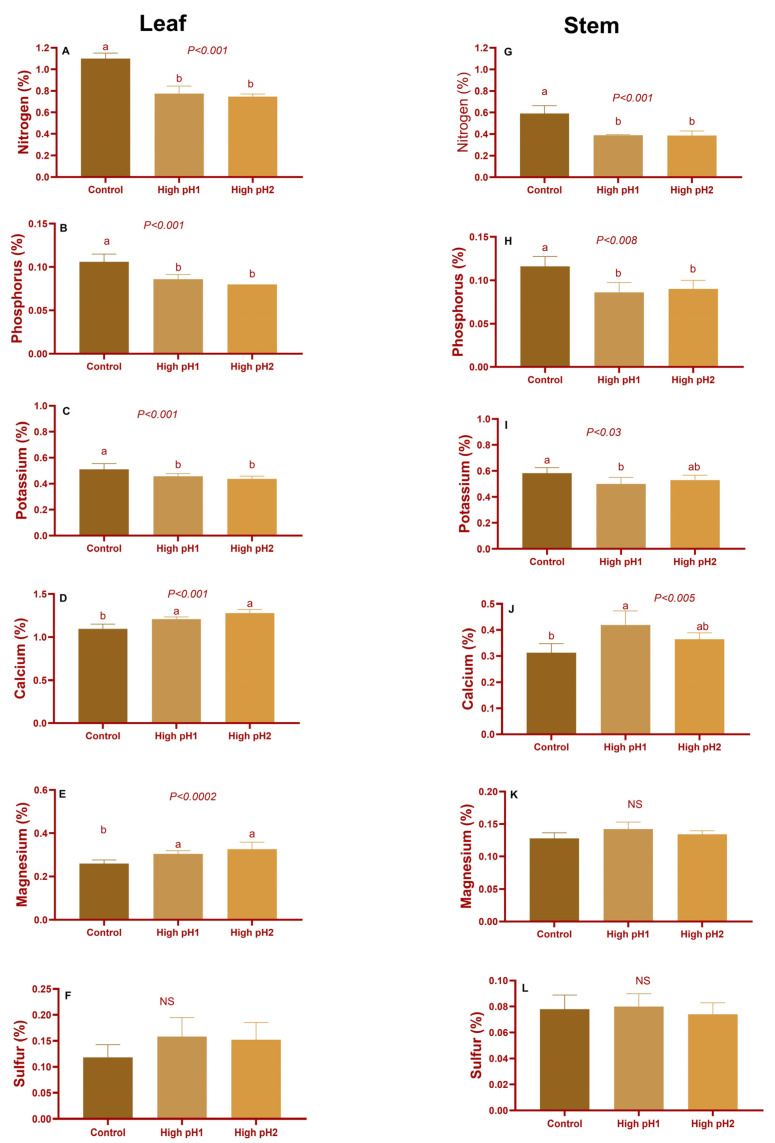
Leaf and stem macronutrients from optimum and two high-pH cranberry pH beds in year 1 (2020). Average ± S.E. macronutrients (**A**,**G**) Nitrogen (**B**,**H**) Phosphorus (**C**,**I**) Potassium (**D**,**J**) Calcium (**E**,**K**) Magnesium and (**F**,**L**) Sulfur in leaves and stems of high-pH and control-pH cranberries. The bars represented with different lower-case letters are significantly different on Tukey-Kramer mode at *p* < 0.05. NS—Nonsignificant.

There were significant differences observed in the macronutrient levels of the leaves and stems collected during the harvest or mature fruit stage in year 1 across the treatments with high soil pH and with control (Figure 5). The concentrations of nitrogen, phosphorus, and potassium in the leaves of plants grown in high pH1 and high pH2 soil were found to be significantly lower (*p* < 0.008 to *p* < 0.001) compared to control plants. The observed decrease in percentage was found to vary between 29% and 34% for N, 18% and 25% for P, and 9% and 15% for K in the leaves and stems of plants grown in high pH1 and pH2 soil conditions (Figure 5A–C,G–I). The concentrations of calcium and magnesium were observed to be elevated in the leaves and stems of plants grown in soil with high pH levels (pH1 and pH2). The increase in calcium content ranged from 10% to 33%, while the increase in magnesium content ranged from 4% to 25%. No statistically significant differences were observed in the levels of sulfur (Figure 5D–F,J–L). In contrast to the observations made in year 1, the levels of sulfur exhibited a significant increase (*p* < 0.001) in soils with high pH compared to the control group of plants (Figure S3F–L).

**Figure 6:**
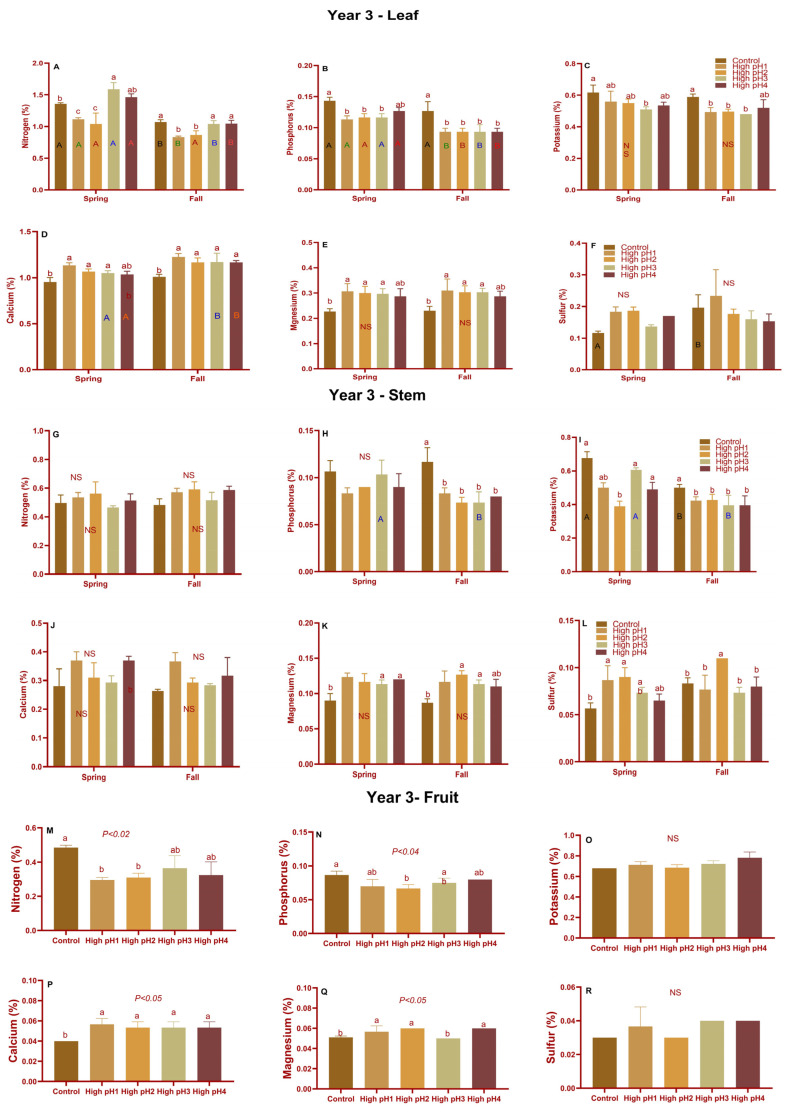
Leaf (**A**–**F**), stem (**G**–**L**), and fruit (**M**–**R**) macronutrients in cranberry plants from four high-pH beds along with optimum-pH beds in year 3 (2022). Average ± S.E. macronutrients (**A**,**G**,**M**) Nitrogen (**B**,**H**,**N**) Phosphorus (**C**,**I**,**O**) Potassium (**D**,**J**,**P**) Calcium (**E**,**K**,**Q**) Magnesium and (**F**,**L**,**R**) Sulfur in high pH and controlled pH cranberries. Samples of stems and leaves were collected in both spring (after bud break) and fall (harvest). According to Tukey’s-Kramer, the bars with different lowercase alphabets are significantly different across treatments within the same season (either spring or fall), whereas the bars with different uppercase letters of the same color are to show significant differences between seasons for the same treatment. The bars of leaves and stems that did not have an upper-case letter did not differ significantly at *p* < 0.05. NS—non-significant.

Comparable substantial variations in macronutrient concentrations were noted during the third year’s harvest, mirroring the findings from both the first and second years. (Figure 6). Nevertheless, there was no significant difference observed in the N and P levels between the leaves and stems of the spring samples (Figure 6A,B,G,H). The remaining macronutrients exhibited a similar trend as in year 1 with regard to their significant increase or decrease in high-pH soils compared to the control for both leaves and stems. In years 1 and 3, when the leaves and stems micronutrients were measured separately, the significant differences, especially sodium, iron, and manganese, were significantly different across high pH and control pH treatments (Tables S3–S5).

#### 2.5.2. Fruit Macro and Micronutrients

**Figure 7:**
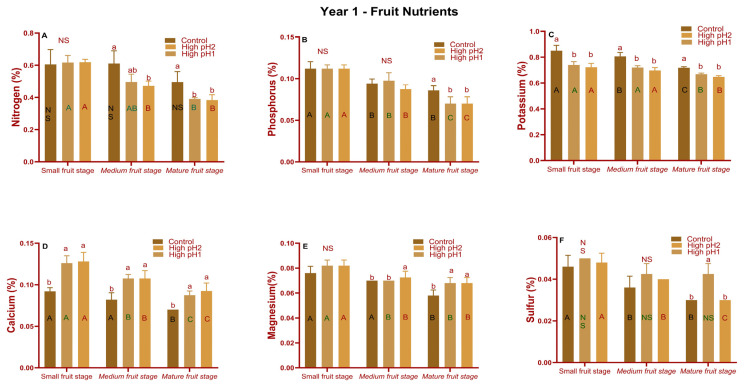
Cranberry macronutrients at three different fruit developmental stages collected from two high-pH and optimum-pH beds in year 1 (2020). Average ± S.E. macronutrients (**A**) Nitrogen (**B**) Phosphorus (**C**) Potassium (**D**) Calcium (**E**) Magnesium and (**F**) Sulfur in fruits of high pH and control pH cranberries. The bars with different lowercase alphabets are significantly different among within each fruit stage, while the bars with different uppercase letters are significantly different among fruit stages within each treatment according to Tukey’s-Kramer at *p* < 0.05. NS means non-significant differences.

The nitrogen (medium and mature fruit), phosphorus (mature fruit), potassium (all three stages), calcium (all three stages), magnesium (medium and mature fruit stage), and sulfur (mature fruit stage) nutrients were significantly different (*p* < 0.05 to *p* < 0.001) across treatments in year 1. N, P, and K were reduced in high soil pH fruit samples, as they were in upright shoot samples. In high pH soil samples, calcium and magnesium levels increased (Figure 7D,E). A similar pattern was observed for macronutrients in year 2 fruit samples (Figure S3M–R). The fruit samples in year 3 followed a similar pattern for N, P, Ca, and Mg as in year 1, with substantial differences (Figure 6M–R).

### 2.6. Shoot and Fruit Metabolite Responses to High Soil pH

**Figure 8:**
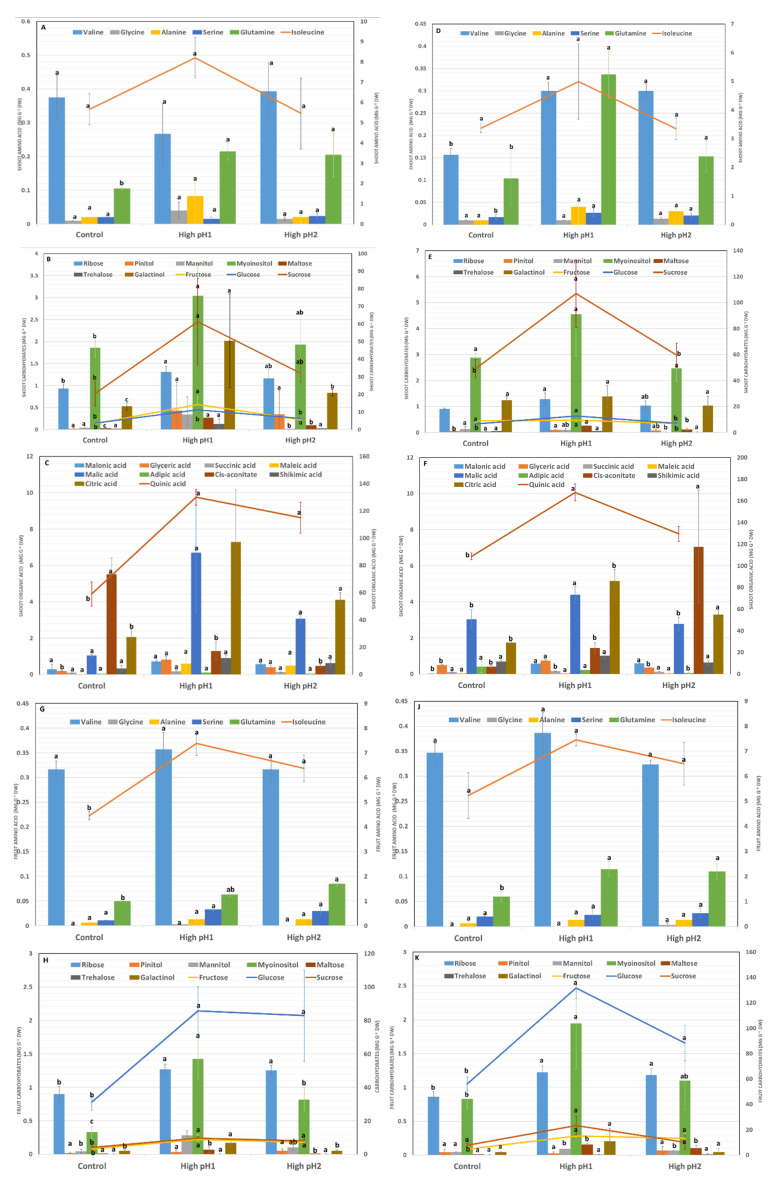

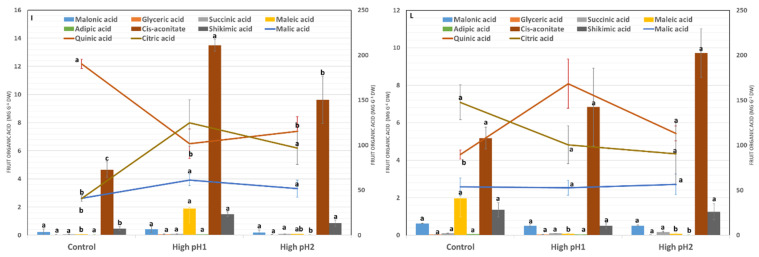
Shoot and fruit metabolites collected at two different time intervals in year 1. Average ± S.E. of shoot metabolites (**A**) Amino Acids (**B**) Carbohydrates, and (**C**) Organic Acids collected in August and (**D**) Amino Acids (**E**) Carbohydrates, and (**F**) Organic Acids in September. Average ± S.E. of fruit metabolites (**G**) Amino Acids (**H**) Carbohydrates, and (**I**) metabolites collected in August and (**J**) Amino Acids (**K**) Carbohydrates, and (**L**) Organic Acids collected in September. According to Tukey’s-Kramer, the bars and lines represented by similar alphabets are not significantly different at *p* < 0.05. The values in the bar graph correspond to the first *Y*-axis, while the lines correspond to the second *Y*-axis.

In this study, a composite of 26 metabolites, consisting of 10 soluble carbohydrates, 10 organic acids, and 6 amino acids, was measured in both upright shoots and fruits harvested during August and October in the year 1 experiment. Among the six amino acids, one compound (glutamine) showed significant variation (*p* < 0.001) in August, while three compounds (valine, serine, and glutamine) displayed differences (*p* < 0.05) in September, with fold increases ranging from 1.9 to 3.2 in shoots. In the month of August, it was observed that the levels of two specific amino acids, glutamine and isoleucine, were higher in fruits grown in high pH soils compared to the control group (Figure 8A,D,G,J).

In August high-pH soil shoot samples, the sugars ribose, fructose, glucose, myoinositol, mannitol, maltose, and galactinol were higher than in controls. Except for myo-inositol and galactinol, the same carbohydrates were reduced in September compared to August samples but remained significantly higher in soil pH samples than in the control, with fold increases ranging from 1.2 to 9.0, with maltose showing the greatest increase (Figure 8B,E). The fold increase in high soil pH fruit samples varied between 1 and 16, with the highest increase observed for maltose (Figure 8H,K). For organic acids, the concentrations of glyceric, cis-aconitic, citric, and quinic acid in shoot samples collected in August displayed variations across treatments (*p* < 0.001). Generally, higher concentrations of these acids were observed in samples with high soil pH, apart from cis-aconitic acid, which showed a reduction (Figure 8C,F).

In the September shoot samples, it was observed that succinic, malonic, and malic acid levels were significantly higher in high pH soil samples compared to control. The fold increase in these organic acids ranged from 1.2- to 39-fold, with malonic acid having the greatest increase. Even though there was an increase in some organic acids in high-pH soil August fruit samples, only quinic acid decreased in September fruit samples. The fold increase ranged from 1.2 to 34, with maleic acid showing the greatest increase and quinic acid the greatest decrease (Figure 8I,L). Glutamine was the only amino acid that consistently differed between control and high pH soil treatments in both shoot and fruit samples (Figure 8).

### 2.7. Soil pH vs. Fruit Traits vs. Fruit Nutrients vs. Fruit Metabolites

Soil pH was negatively correlated with fruit size during the first and third years of the study. Although only a limited number of statistically significant correlations were identified (Table S5, Figure S4), most macronutrients in cranberry exhibited a negative association with soil pH, with the exception of calcium and magnesium in fruits and shoots. Correlations between nutrients and metabolites—including carbohydrates, amino acids, and organic acids—were largely inconclusive; however, macronutrients nitrogen, phosphorus, and potassium showed positive relationships (Table S5, Figure S4L).

In August of the first year, soil pH was positively related to fruit calcium and negatively related to fruit size, maleic acid, malic acid, and quinic acid (Table S6, Figure S4). Fruit alanine, glutamine, ribose, and fructose were positively correlated to soil pH. Nitrogen, potassium, maleic acid, and citric acid were positive. In September of year 1, soil pH negatively affected fruit size, phosphorus, succinic acid, and malic acid. Soil pH was positively correlated with fruit alanine, serine, ribose, fructose, and glucose (Figure S4E–I).

In year 3, the soil pH was negatively correlated with fruit size, fruit number, stem phosphorus, stem potassium, leaf phosphorus, leaf potassium, and fruit nitrogen. Nonetheless, it was inversely related to leaf Ca, leaf Mg, stem Mg, and fruit Ca. The size of the fruit correlated positively with leaf P, leaf K, stem K, and fruit N, and negatively with leaf Ca, leaf Mg, and fruit Ca. There was a significant correlation between leaf P, stem K, and fruit N. However, Ca and Mg were negatively correlated with N, P, and K in stems, leaves, and fruits. Ca and Mg also inhibit the production of maleic acid, malic acid, quinic acid, glyceric acid, and citric acid in fruits. Ca was negatively correlated with alanine, glutamine, serine, glucose, fructose, and ribose in fruit (Figure S4).

**Figure 9:**
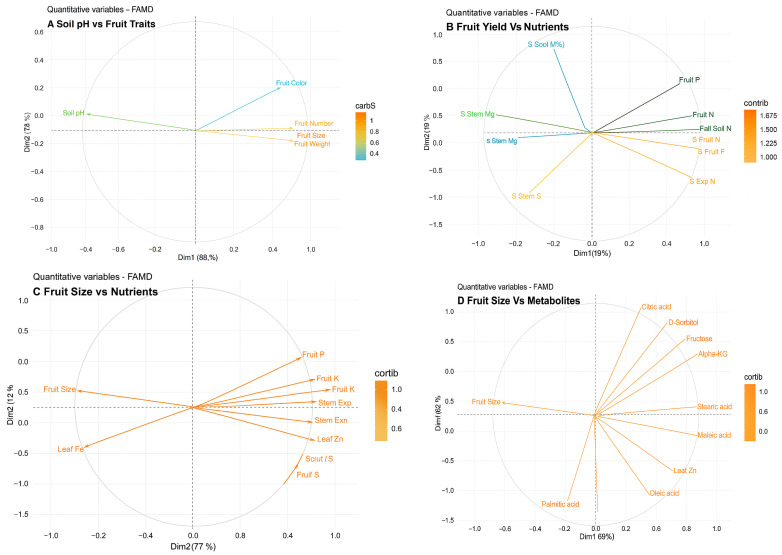
Quantitative variables of the first two dimensions resulting from factor analysis of mixed data (FAMD) of (**A**) Soil pH vs. Fruit traits (**B**) Fruit yield vs. Nutrients (soil, plant, and fruit) (**C**) Fruit size vs. Nutrients (soil, plant, and fruit) and (**D**) Fruit size vs. Metabolites (Shoot and Fruit). Plot shows the top 15 contributing factors. In (**B**) “S” in front of the nutrient name represents Spring. The dataset D includes data from 2020 (year 1) since the metabolites were measured only in that experimental year.

The FAMD results indicate that soil pH significantly influences fruit yield and size more than fruit color and firmness (Figure 9A). This is consistent with results analyzed through ANOVA and correlation matrix analysis. Soil pH significantly influenced all experimental variables in the FAMD analysis, revealing that in dim 1, fall soil C, fruit N, leaf P, and Fe levels in both stem and leaf were the most impacted. Dim 2 is characterized by significant contributions from fall soil B, spring soil N, leaf N, soil B, and leaf S (Figure S5A).

The top contributors of nutrients in fruit number in dim 1 were fruit N, fall soil C, spring stem Fe, leaf Fe, leaf P, and fruit Ca. In dimension 2, the top contributors were spring Nitrogen in the soil, Copper in the leaves, Boron in the stem, and Sulfur in the leaves (Figure 9B). The main metabolites affecting fruit size under the influence of soil pH in dim 1 were shoot ribose, serine, quinic acid, pintol, and maltose. In dim-2, the key contributors were shoot mannitol, cis-aconitate, and glycine (Figure 9D). We integrated nutrient and metabolite variables to analyze their impact on fruit size together. Most metabolites were categorized under dim 1, with fruit malic acid, fruit sucrose, leaf S, fruit serine, and fruit fructose being the primary contributors. Dim 2 comprises mainly of nutrients such as stem S, fruit P, fall soil Fe, shoot galactic acid, and fall soil Zn (Figure S5B).

## 3. Discussion

### 3.1. Effect of Soil pH on Soil Nutrient Availability and Plant Nutrient Absorption in Cranberries

The pH value commonly measured in soil studies does not represent the pH of the solid phase itself, but rather that of a solution in equilibrium with the soil. Soil pH is typically determined by measuring the hydrogen ion concentration in a liquid extract that has been brought into contact with the soil matrix. The result is influenced by both the solution used and the net charge of the soil [22]. The current study consists of four high-pH beds with soil pH ranging from 7.0 to 7.2 measured over a period of four years. Elevated irrigation water alkalinity (pH ~8.0) likely drove the soil pH upward, a common phenomenon in irrigated systems where bicarbonate-rich water increases soil alkalinity. Various soil conditions, including moisture, type and structure, organic matter, and soil pH, have a significant impact on the availability of nutrients along with biological soil properties. Typically, the root system of fruit trees exhibits a broader range of soil pH suitability for the uptake of macro elements. However, the availability of micronutrients is closely linked to the soil pH [23]. Increase in sulfate availability increases with higher pH due to decreased sorption by soil [16], and also the addition of granular sulfur to reduce soil pH in cranberry in high pH soils might have caused no significant differences in sulfur levels compared to control (Figure 3, Figure 4, Figure 5 and Figure 6 and Figure S3). The lack of sulfur differences across treatments may reflect external management (sulfur application) rather than a true soil pH effect, highlighting the importance of distinguishing fertilization effects from pH-driven solubility changes.

The possible reason for the higher amounts of nitrogen might be due to the presence of the nitrate form of nitrogen in irrigation water that high pH3 and high pH4 beds received (Table S1). This suggests that nitrate from irrigation water can supplement plant N status, but cranberries generally prefer ammonium (NH_4_^+^). Excess nitrate under alkaline conditions could therefore explain poor growth despite adequate total N levels [20,24,25]. Nitrogen mineralization increases along with increasing pH, but the uptake of ammonium ions decreases with reduced pH [22]. Cranberries grow in acidic soils and prefer the ammonium form of nitrogen. Nitrate is the predominant N-source in soils under high pH, while NH_4_^+^ is at low pH due to low vitrification rates in acidic soils. At high soil pH with the presence of many nitrification bacteria, the ammonium form of N will be converted into nitrate form, especially in high pH1 and high pH2 soils [24]. Cranberries produce and thrive more effectively when supplied with NH_4_-N than NO_3_-N. In general, their growth is suboptimal when provided with the NO_3_ form of nitrogen [25], which suggests the poor performance of cranberry despite the N availability in high-pH soils. We did not analyze the amount of nitrate and ammonium forms of nitrogen in plants and soils, focusing solely on total nitrogen in this study.

When evaluating the impact of pH on availability, two crucial factors to consider are the influence of pH on the desorption rate and its effects on the uptake rate by plant roots [26]. The phosphate uptake by plant roots increases as the pH reduces, which might have decreased phosphorus uptake in cranberry plant tissue under high soil pH. High pH soils often precipitate phosphorus as calcium phosphates, reducing the soluble pool. This mechanism explains why total soil P may remain similar, yet plant tissue P declines [27]. The process of potassium uptake includes the export of protons, and magnesium follows a similar mechanism. High pH therefore promotes both magnesium and potassium uptake [28,29]. The discrepancy between adequate soil K and reduced tissue K likely reflects impaired proton-mediated uptake under alkaline conditions. Excess Ca^2+^ and Mg^2+^ in high-pH soils may also compete with K^+^ uptake channels, compounding the deficiency [11,25,30]. In contrast to K, the concentration of Mg was found higher in all plant samples following their mechanism of increased absorption at high soil pH [22]. This pattern aligns with the strong solubility of Mg under alkaline conditions and its preferential uptake relative to K when both ions are present [30].

The current study revealed elevated concentrations of calcium and magnesium in high-pH soils and in the corresponding plants and fruits. Elevated Ca and Mg uptake is consistent with increased cation exchange capacity under high pH, but excessive accumulation may contribute to nutrient imbalances, particularly antagonism with K and micronutrients [28,29,30]. Micronutrient responses were variable across years, but the general trend of reduced Fe, Mn, Zn, and Cu under alkaline conditions is consistent with reduced solubility and chelation at high pH. This mechanism likely contributes to observed growth limitations [5,9,29,30].

### 3.2. Photosynthesis and Chlorophyll Fluorescence Were Affected by Soil pH in Cranberries

The survival and adaptability of cranberry plants to high pH soil stress are dependent upon physiological responses. These stress conditions can detrimentally affect the growth and development of plants by influencing various physiological processes [14]. High soil pH stress has been observed to result in several important responsive mechanisms and physiological implications in cranberry plants. Impaired nutrient uptake (particularly N and P) likely reduces photosynthetic efficiency, while stress-induced metabolite shifts reflect compensatory carbon allocation under high pH stress [20,21].

Photosynthesis is a crucial physiological process in plants, wherein photosynthetic products serve as fundamental materials and energy sources for all organisms. Similar to the blueberry study conducted by [14], the current study in cranberries also found a reduction in photosynthesis, water use efficiency, and A/Ci. In ecological studies, it was discovered that foliar N and/or P are positively correlated with the photosynthetic rate at regional and global scales [31]. The potential decline in photosynthetic activity could possibly be attributed to a decrease in leaf nitrogen (N) and phosphorus (P) concentrations, which are essential factors influencing gas exchange processes [32]. This suggests that nutrient limitation, rather than direct pH toxicity, underlies much of the photosynthetic suppression in cranberries. Lower tissue N reduces Rubisco activity, while reduced P constrains ATP and NADPH regeneration, both critical for CO_2_ assimilation [32].

Chlorophyll fluorescence in plants has been used to assess environmental stress [33]. The observed declines in Y(II) and ETR in alkaline soils have detrimental implications for the reaction center of photosystem II (PSII) and energy transfer processes, which may be associated with compromised nutrient uptake [34]. Declines in Y(II) and ETR reflect impaired efficiency of PSII electron transport, possibly due to disruption of thylakoid membrane integrity under nutrient stress. In particular, Mg^2+^ imbalances at high pH could alter chlorophyll structure, further destabilizing energy transfer [34]. The parameter Fv/Fo is widely recognized as a metric for assessing the abundance and magnitudes of functional photosynthetic reaction centers. Reduced Fv/Fo under alkaline stress likely indicates damage or loss of PSII reaction centers, similar to responses seen under salinity or pathogen stress, reinforcing the interpretation that high pH compromises core photosynthetic machinery. The decline in Fv/Fm, a proxy for maximum photochemical efficiency, suggests chronic photoinhibition. This is consistent with nutrient-limited plants being unable to balance light capture and biochemical energy use, leading to sustained damage to PSII [34,35,36].

### 3.3. Shoot and Fruit Metabolite Changes in Response to High pH Stress

The biosynthesis, transport, and storage of numerous primary and secondary metabolites are modified by abiotic stresses [37] such as high soil pH. To keep energy and metabolism in balance, plants rewire their central metabolism in response to abiotic stresses [29]. In cranberries, the consistent alteration of amino acids, sugars, and organic acids under high pH stress suggests a coordinated metabolic shift aimed at buffering nutrient imbalances and supporting osmotic adjustment, as seen in other stress-adapted plants [38,39].

Several plant stress responses are controlled by metabolites that are part of the plant’s central metabolic network [40]. Several studies have demonstrated that the total amino acid in plants is positively correlated with the N, P, and K uptake [41]. Amino acids play a crucial role in protein synthesis and additionally function as precursors for a diverse range of metabolites that contribute to various aspects of plant growth and response to different stressors [42,43]. Glutamine is important in nitrogen translocation and storage in many plants. The increased levels of glutamine might be related to compensating for the mineralization of N under high soil pH conditions [22]. Elevated glutamine levels may reflect an adaptive mechanism where excess nitrate (formed under alkaline soils) is assimilated into amino acids for detoxification and redistribution, a role noted in stress physiology across crops [38,44]. This amino acid appears to be involved in the mineralization of nitrogen and mitigates environmental stresses via facilitating the accumulation of stress tolerance components such as sucrose, proline, and chlorophyll, among others [44]. Similar to cranberries, the increase in soluble sugars in response to alkali has been observed in different studies [42,43], indicating that plants subjected to alkali stress were prepared for maintaining sustained growth and acquiring carbon under stress conditions. The sugar accumulation in response to environmental stress helps in mitigating oxidative stress by producing different stress components such as reactive oxygen species through sugar signaling [42,43]. The accumulation of sugars accompanied by a decrease in nutrient absorption under high soil pH stress is likely attributable to the decreased use of sugars in the actively growing tissue, reducing growth, yield, and fruit size.

Numerous studies have demonstrated that different plant species possess the ability to produce substantial quantities of organic acids in response to alkali stress, serving as a buffering mechanism, enabling plants to withstand fluctuations in their surroundings, while simultaneously preserving intracellular pH equilibrium and ion homeostasis [43]. Most plants increase the concentrations of organic acids to mitigate the effects of high pH, but the nature of this accumulation varies by species [18]. The temporal dynamics of organic acids in cranberries likely reflect both developmental programming and stress-induced regulation. Under alkaline stress, organic acids such as malate and citrate not only buffer cytosolic pH but also chelate excess cations (e.g., Ca^2+^, Mg^2+^), thereby maintaining ion balance [45].

According to previous studies, plants cultivated in alkaline soils release citric acid and malate through their root systems [46]. This mechanism facilitates the absorption of vital nutrients such as phosphorus and iron by reducing the pH levels in the rhizosphere [37], and another study indicates that the application of a 5% malic acid treatment resulted in an increase in soil ammonium-nitrogen content and a decrease in soil nitrate-nitrogen content [47]. Even though the current study did not examine the roots and nitrogen form, the accumulation of citric acid and malic acid in both shoots and fruits might be related to ameliorating the effects of high soil pH and to reducing the pH levels. The components of metabolites involved in the shikimate pathway, such as quinic acid and shikimic acid, were significantly increased. Organic acids such as malic, citric, oxalic, and quinic acids—play critical roles in plant responses to abiotic stresses like salinity, drought, temperature fluctuations, and heavy metal toxicity and contribute to stress mitigation [48]. However, the quantity of quinic acid is important for cranberry as a chemical defense compound and for its nutrient properties [48,49]. Elevated quinic acid in cranberries under alkaline stress may thus serve a dual role: maintaining metabolic flux through the shikimate pathway for phenolic biosynthesis and acting as a defensive metabolite against stress-induced damage [48].

### 3.4. Interactions of Plant Nutrients and Metabolites Under High Soil pH and Overall Reduction in Fruit Traits

The ripening process of cranberry fruit has been associated with various physiological alterations, including an augmentation in fruit size, pericarp area, and locule size. Additionally, there is an elevation in the levels of sugars and phytochemical compounds, such as anthocyanins and flavanols [50]. The cranberry fruit’s metabolite composition can exhibit variability due to factors such as environmental conditions (Table S5, Figure S4). Such physiological and metabolic shifts provide the biochemical foundation for fruit quality traits, linking nutrient availability and metabolite accumulation to final yield, size, and firmness [51]. Our integrative analysis shows that macronutrients, especially N and P, strongly underpin fruit size and firmness. At the same time, sugars appear to be the dominant metabolite class associated with fruit size, consistent with their dual role as structural carbon sources and osmotic regulators [52]. The most important factor affecting soluble sugar content is total soil nitrogen. According to previous studies, adequate nitrogen application promotes the accumulation of soluble sugar [53] and regulates the conversion and transfer of soluble sugar in plants [54,55] and influences organic acids and amino acids [41,43]. Comparable findings in tomatoes show that balanced N, P, and K inputs improve soluble sugar and organic acid metabolism, underscoring the role of soil fertility management in shaping fruit quality [30,54]. Hence, the metabolites were influenced by the availability of the nutrient elements, and the limitation in plant nutrient absorption of elements due to high soil pH stress possibly resulted in the regulation of metabolites. Further, it is worth noting that soil C was the most important factor influencing fruit size and yield as soil pH increased (Figure 9). Soil organic carbon is crucial in determining the availability of nutrients in the soil and has a significant impact on the delivery of nitrogen and availability of phosphorus, iron, and copper. Enhancing soil organic carbon levels is widely recognized as a key factor in boosting yields while minimizing adverse consequences under stressful environments [56,57]. Organic matter improves cation exchange capacity, buffers pH fluctuations, and provides a slow-release source of nutrients [58], explaining why high soil organic carbon enhances resilience under stress conditions. This implies that overall, soil factors may have been more important in nutrient absorption and determining fruit yield and size.

Fruit calcium emerged as a significant factor in the top list of FAMD analysis, influenced by high soil pH and impacting fruit size, when considering nutrients alone and together with metabolites (Figure 9 and Figure S5). One important role of Ca^2+^ in plants is to maintain the integrity of cell membranes and the strength of the cell wall in plants [59]. Calcium ions can form cross-links between polysaccharides present in the cell wall, particularly pectins, thereby exerting an influence on the extensibility of the cell wall. In this study, calcium accumulation was significantly related/influenced nine metabolites’ concentrations in fruits. Elevated Ca^2+^ may also alter metabolic signaling cascades, influencing carbohydrate partitioning and organic acid biosynthesis. Beyond pectin cross-linking, Ca^2+^ interactions with proteins and other wall components can stiffen tissues, reducing expansion potential [60]. An excessive concentration of calcium ions has the potential to enhance the rigidity of the cell wall, thereby restricting its expansion [60], which might have resulted in reduced fruit size. This highlights a classic trade-off: while Ca^2+^ is critical for fruit firmness and shelf life, excessive accumulation under alkaline soils may compromise growth and yield [60].

## 4. Materials and Methods

### 4.1. Selection of High pH Soil and Control pH Soil Beds

Cranberries are cultivated in specialized beds where the soil is predominantly sandy, enriched by the natural accumulation of organic matter, and maintained through the annual addition of approximately 1 cm of fresh sand. These beds typically range in size from 3 to 4.5 acres and support a dense, continuous mat of perennial vines rather than discrete individual plants. Cranberry vines produce two types of shoots: runners, which grow horizontally and spread across the bed, and uprights, which grow vertically to a height of up to 15 cm. Uprights are further classified as either vegetative, supporting new growth, or fruiting, which bear the crop. Because the vines interweave and expand over time, production is measured at the bed level, generally expressed per unit area (e.g., square feet), rather than by individual plants. In this study, two high-pH beds and one control bed were sampled during the 2020 and 2021 growing seasons. In 2022, two additional high-pH beds were added, bringing the total number of experimental beds to five. The beds included in this study had a root soil depth of approximately 15–17 cm and contained vines that were 15–20 years old, consistent with the age range of commercial cranberry production systems.

In years 1 and 2, there were three experimental beds (two high-pH and one control), while in year 3 the number increased to five beds, consisting of four high-pH beds and one control. Several criteria were considered when selecting the high-pH beds, including soil pH history (over five years), location, bed age, cultivar, and management practices. All four high-pH beds maintained a minimum soil pH of 6 throughout the growing season, even after applications of granular sulfur. The five experimental beds were located in central Wisconsin, were in close proximity to one another, were of similar age (>10 years), and were all planted with the cultivar Stevens. Due to their proximity, the beds were exposed to similar weather conditions (Table 1 and Table S2). However, irrigation water for the high-pH and control beds originated from different water sources, and the differences in soil pH were primarily attributable to variation in irrigation water pH (Table 1 and Table S1).

Fertilization practices across the cranberry beds were largely uniform, with the exception of granular sulfur applications. High-pH beds were typically treated with 50–68 kg per acre of granular sulfur, applied at four to five intervals during the growing season (May to October) to mitigate elevated soil pH. The control bed also received approximately 50 kg per acre of granular sulfur, but at three intervals per growing season.

### 4.2. Sample Collection

The high-pH cranberry beds and the control bed each consist of an area of approximately 4.5 acres. To account for potential microclimatic variation and to better assess the impacts of high soil pH, each bed was subdivided into six zones (n = 6). From each zone, five to six soil cores were systematically collected to a uniform depth of 15 cm using a soil sampler. The cores from each zone were then combined and thoroughly mixed in a bucket to ensure homogeneity, and each bucket of homogenized soil (derived from five soil cores) was treated as a single replicate sample. For plant tissue sampling, five upright shoots (both vegetative and fruiting) were collected at random from ten different locations within each zone and pooled to form one replicate sample, which was subsequently used for nutrient quantification. To evaluate fruit traits, a square frame measuring 0.092 m^2^ (constructed from PVC pipe) was placed randomly within each zone, and all fruits contained within the frame were collected and measured for area, length, width, number, color, and firmness.

### 4.3. Sampling Times

In the first year of the study, upright shoots and fruits were collected at three-time intervals from two beds with high soil pH levels and one control bed. In July, the initial collection of soil and fruit samples was conducted to assess parameters such as soil pH, soil nutrient composition, and fruit traits. In August, the subsequent batch of soil, upright, and fruit samples were collected for assessing soil pH levels, nutrient content, and metabolite concentrations. At the end of September, a third set of samples was obtained, coinciding with the maturation of the fruits. The collected samples include a combination of vegetative and reproductive shoots, with the stems and leaves being separated for further analysis. During the second year of the study, samples of soil and plants were collected at the end of the growing season, specifically during the mature fruit stage, in the first week of October. The vegetative and fruiting upright samples were collected separately for nutrient analysis.

In the third year, soil and upright samples were collected in the spring after bud break to determine the nutrient content of both at the start of the growing season. In October, soil, upright shoots, and fruit samples were collected during harvest (mature fruit stage). The fruit stages described here were determined by observing the stages of control plants.

### 4.4. Soil, Water, and Plant Nutrient Analysis

The soil pH for each replicate was measured in a soil sample paste combining 1:2 soil using an ECe/pH meter (Ino LAB pH/Cond 720, WTW series, Weilheim, Germany). Organic matter and rocks were removed from the soil samples using a 2-mm sieve after they were dried in a dryer. The soil nutrients (P, K, Ca, Mg, S, Cu, B, Na, Fe, Mn, and Zn) were measured using the Mehlich 3 extractable method at the Agricultural Diagnostic Laboratory, University of Arkansas, Fayetteville, AR. The content of elements in plants and fruits (P, K, Ca, Mg, S, Cu, B, Na, Fe, Mn, and Zn) was measured at the same laboratory using acid digestion by inductively coupled plasma (ICP). Total nitrogen and carbon in soil, upright leaves and stems, and fruits were determined by combustion using a Carlo-Erba elemental analyzer (CE Elantech EA1112, Lakewood, NJ, USA). Irrigation water samples were collected from reservoirs (the inlet water sources) of one control bed and four high-pH beds at the beginning (spring, May) and end (fall, September) of the third growing season. These water samples were analyzed for elemental composition at AgSource Laboratories, WI, USA.

### 4.5. Gas Exchange Parameters and Chlorophyll Fluorescence

Gas exchange and chlorophyll fluorescence were assessed during the third year of the study in the months of June (early bloom) and September (mature fruit). In each of the six zones, three to four upright shoots were used for measuring transpiration, photosynthesis, internal carbon supply, and stomatal conductance using the CIRAS 3, a CO_2_/H_2_O gas analyzer, and a conifer cuvette (PP system, MA, USA). The leaf area of the upright samples was estimated using a scanner and the Wincam NDVI software (https://regentinstruments.com/assets/wincam_about.html, accessed on 25 July 2025) developed by Regent Instrument, CA. The gas exchange parameters were recalculated in accordance with the leaf area. The chlorophyll fluorescence parameters Fv/Fm and Fv/Fo were assessed by using an Fv/Fm meter in accordance with the guidelines provided in the instruction manual (Opti-sciences, Inc., Hudson, NH, USA). The Y(II) or delta Fv/Fm and ETR parameters were measured using a Y(II) meter manufactured by Opti-Sciences, Inc., located in Hudson, NH, USA. The parameters measured were Fv/Fm: Maximum photochemical efficiency of PSII Fv/Fo: A more sensitive detector of stress than Fv/Fm, but it does not measure plant efficiency or correlate to photosynthesis measurements. Fo: Minimum fluorescence Fm: Maximal fluorescence.

### 4.6. Fruit Traits

Fruit firmness: The maximum compression force and maximum compression distance were measured for each zone replicate at each time point of collection, with a total of 35–40 fruits per zone replicate. The measurements were performed on a Texture Analyzer (TA.XTPlus Connect, Textural Technologies, Hamilton, MA, USA) operating at a test speed of 1 mm·s^−1^. The target mode was configured to “Strain” at a level of 30%. The attributes of maximum compression force and maximum compression distance are correlated with the apex of the curve and the corresponding distance at which this peak force is reached, respectively, within the context of a particular compression profile [50]. The fruit area, length, and width of all fruit samples collected from each 0.09 m^2^ area were measured with a scanner and Regent instrument, CA’s wincam NDVI software. The number of fruits was counted per 0.09 m^2^ area and weighed on a balance to determine the weight in g. The total anthocyanin content, or fruit color, was estimated using the total anthocyanin content (TACY) method, as described in the previous article Diaz-Garcia et al., 2019 [50].

### 4.7. Metabolite Measurements

In year 1, upright shoot and fruit samples for metabolite analysis were collected as described in Section 2.2 during the months of August and September in the first year. The samples were collected in the field, placed on dry ice, transported to the lab, and stored at −80 °C until further analysis. The samples were freeze-dried and ground into a fine powder using a mortar and pestle. In this study, a quantity of 60 mg of dry weight was subsequently subjected to pulverization using a TissueLyser II bead mill (Qiagen, Valencia, CA, USA). The pulverization process was carried out in 2 mL SealRite microcentrifuge tubes (USA Scientific, Ocala, FL, USA), which were equipped with a ceramic bead measuring 6.4 mm in diameter (MP Biomedicals, Santa Ana, CA, USA). The soluble metabolites were extracted using an aqueous methanol solution, and their quantities were assessed employing gas chromatography coupled with mass spectrometry, in accordance with the methodology described in the previous study [61]. Standard curves were generated using four-point curves and known concentrations of soluble sugars, sugar alcohols, organic acids, and amines. These standard curves were then used to calculate the concentration of these compounds in the samples.

### 4.8. Statistical Analysis

The data for statistical analysis were subjected to a one-way (ANOVA)—Tukey’s-Kramer by using Graph pad prism 7.0 to understand the high soil pH effect on nutrients, metabolites, fruit traits, gas exchange, and chlorophyll fluorescence parameters. Two-way ANOVA was used to understand the effect of soil pH and different sampling timings. The impact of high soil pH and its relation to nutrient uptake and metabolite changes was studied using a correlation matrix (Graph pad Prism 7.0). We examined the effects of high pH on various soil, plant, and fruit characteristics by analyzing soil pH, nutrients in soil and plant tissues, yield, fruit traits, and shoot and fruit metabolites using Factor Analysis of Mixed Data (FAMD) with the FactoMineR package 2.12 in R. This approach enabled us to explore relationships between numerical and categorical variables concurrently. The statistical analyses were conducted using R, version 4.2.2.

## 5. Conclusions

This study represents the first comprehensive investigation into the effects of soil pH on cranberry growth and fruit characteristics and its interaction with nutrient dynamics and metabolite profiles. Our findings clearly show that elevated soil pH disrupts cranberry performance at multiple levels, reducing vegetative growth, yield, fruit size, color, and firmness through a combination of nutrient imbalances and impaired physiological processes. High soil pH constrained photosynthesis by reducing electron transport and Photosystem II efficiency, likely linked to nitrogen and phosphorus deficiencies, which in turn limited carbon assimilation and plant productivity. Macronutrient concentrations—specifically nitrogen, phosphorus, and potassium—in leaves, stems, shoots, and fruits were negatively associated with soil pH. In addition, elevated pH altered the balance of key metabolites, with amino acids, soluble sugars, and organic acids reprogrammed as part of stress-adaptation pathways. This metabolic shift reflects the plant’s attempt to buffer nutrient stress but was insufficient to prevent yield losses. Calcium and magnesium concentrations were positively correlated with high soil pH and inversely related to fruit size. Excess Ca and Mg accumulation under alkaline conditions further antagonized K and micronutrient uptake, reinforcing the negative impacts of high soil pH on fruit development and size. Overall, this study highlights that high soil pH impairs cranberry growth not simply by reducing nutrient availability, but by triggering a cascade of physiological and metabolic disruptions. These insights provide a basis for developing targeted management strategies, including the use of soil acidification amendments, optimized fertilizer formulations (favoring ammonium-based N and soluble P), and practices that enhance soil organic carbon. Moreover, understanding the physiological and biochemical components associated with fruit traits and pH stress responses can help identify practical solutions for cranberry growers facing these challenges. Future research should also explore breeding or rootstock selection for greater tolerance to alkaline soils.

## Figures and Tables

**Table 1:** Irrigation water pH, soil pH, and soil elements in control and four high soil pH cranberry beds.

	Control	High pH1	High pH2	High pH3	High pH4
**Water pH**	6.0 ± 0.05 b	8.2 ± 0.07 a	8.2 ± 0.07 a	8.1 ± 0.09 a	8.3 ± 0.04 a
**Soil pH**	4.8 ± 0.04 b	7.2 ± 0.09 a	7.2 ± 0.09 a	7.0 ± 0.07 a	7.2 ± 0.06 a
**Organic Matter (%)**	1.9 ± 0.002 a	0.9 ± 0.001 b	0.8 ± 0.002 b	0.3 ± 0.0009 b	0.5 ± 0.001 b
**Available Phosphorus**	38 ± 4.0 a	14 ± 2.8 b	20 ± 2.2 b	-	-
**Total Phosphorus**	40 ± 2.1 a	22 ± 5.4 b	33 ± 4.1 a	37 ± 3.4 a	30 ± 3.2 b
**Potassium (ppm)**	41 ± 3.2 b	24 ± 2.8 c	26 ± 2.2 c	54 ± 3.2 a	34 ± 3.1 bc
**Calcium (ppm)**	77 ± 12.1 c	389 ± 14.5 ab	515 ± 10.9 a	258 ± 12.3 ab	405 ± 11.9 ab
**Magnesium (ppm)**	50 ± 1.5 b	102 ± 3.1 ab	155 ± 4.5 a	78 ± 4.7 ab	135 ± 2.1 a
**Sodium (ppm)**	4.8 ± 0.5 b	9.0 ± 2.2 a	9.0 ± 2.1 a	3.9 ± 0.5 b	5.2 ± 0.9 ab
**Sulfur (ppm)**	4.0 ± 0.20 b	7.0 ± 0.50 a	10.0 ± 1.90 a	4.3 ± 0.20 b	3.5 ± 0.50 b
**Iron (ppm)**	65 ± 12.20 bc	55 ± 10.05 bc	42 ± 18.23 c	87.2 ± 16.59 b	160 ± 12.57 a
**Manganese (ppm)**	6 ± 0.50 a	2 ± 009 b	3 ± 0.05 b	0.8 ± 0.005 c	7 ± 0.02 a
**Zinc (ppm)**	1.0 ± 0.12 b	0.6 ± 0.05 b	0.6 ± 0.04 b	1.4 ± 0.15 b	4.4 ± 0.12 a
**Copper (ppm)**	0.3 ± 0.001 b	3.1 ± 0.001 a	1.1 ± 0.005 b	0.7 ± 0.03 b	2.8 ± 0.02 b
**Born (ppm)**	0.2 ± 0.005 a	0.2 ± 0.001 a	0.1 ± 0.00 a	0.2 ± 0.005 a	0.4 ± 0.008 a
**Nitrogen (%)**	0.03 ± 0.009 a	0.02 ±0.001 b	0.01 ± 0.003 b	0.03 ± 0.006 a	0.03 ± 0.001 a
**Carbon (%)**	1.09 ± 0.18	0.41 ± 0.11	0.37 ± 0.12	0.56 ± 0.16	0.57 ± 0.11
**Potassium (%)**	4.0 ± 0.15 ab	2.1 ± 0.25 ab	1.7 ± 0.09 b	6.9 ± 0.40 a	3.0 ± 0.20 ab
**Magnesium (%)**	16 ± 1.85 b	29.3 ± 2.25 b	32.3 ± 0.86 a	30 ± 1.22 b	34 ± 1.47 a
**Calcium (%)**	53 ± 2.58 b	67.3 ± 6.30 a	65 ± 1.28 a	61 ± 1.18 a	63 ± 1.75 a
**Hydrogen (%)**	26.7 ± 2.50 a	0	0	2.08 ± 0.54 b	3.75 ± 0.74 b
**Sodium (%)**	-	1.3 ± 0.005 a	1.0 ± 0.00 a	-	0.1 ± 0.00 a

Average (3 years) ± S.E. of different soil parameters and nutrients along with irrigation water pH in one optimum pH bed and four different high soil pH cranberry beds. Based on the Tukey-Kramer model, the values represented by similar lowercase alphabets are not significantly different at *p* < 0.05.

## Data Availability

Data will be available upon request.

## Supplementary Materials

The following supporting information can be downloaded at: https://www.mdpi.com/article/10.3390/plants14182833/s1, Table S1: Water analysis data of irrigation water supplied to control pH and four high soil pH beds; Table S2: Soil macro and micronutrients from different soil pH cranberry beds in year 3 during spring (after bud break-initial growing stages) and fall (mature fruit stage); Table S3: Average ± S.E. of different micronutrients in leaves and stems collected in September and fruits at different maturity stages of control and two high soil pH cranberry beds in year 1; Figure S1: The mean ± standard error (SE) of fruit length and width of fruits grown under both control and high soil pH conditions; Figure S2: Mean ± SE of photosynthesis, transpiration, stomatal conductance, and water use efficiency of cranberry uprights measured during the first week of June 2022 (year 3); Figure S3: Mean ± SE of macronutrients of cranberry vegetative and fruiting uprights in year 2. Table S4: The mean ± standard error (SE) of micronutrients s in cranberry vegetative, fruiting uprights, and fruits collected during harvest/mature fruit stage in year 2; Table S5: The mean ± standard error (SE) of micronutrients in cranberry upright leaves and stems collected in fall and spring and fruits collected during harvest/mature fruit stage in year 3; Figure S4: Correlation matrix of soil pH, fruit traits, nutrients, and metabolites of cranberry shoots and fruits in years 1 and 3; Table S6: Correlation matrix of soil pH, fruit traits, fruit nutrients, and fruit metabolites of cranberry fruits in years 1 and 3; Figure S5: Quantitative variables of the first two dimensions resulting from factor analysis of mixed data (FAMD) of (A) Soil pH vs. nutrients (B) Soil pH vs. Nutrients and Metabolites.

## Abbreviations

A	Photosynthesis
ANOVA	Analysis of Variance
CIRAS 3	CO_2_/H_2_O Gas Analyzer
E	Transpiration
ECe	Electrical Conductivity of the Saturation Extract
ETR	Electron Transport Rate
FAMD	Factor Analysis of Mixed Data
Fm	Maximum Fluorescence
Fo	Minimum Fluorescence
Fv/Fm	Maximum Photochemical Efficiency of Photosystem II (PSII)
Fv/Fo	Indicator of Plant Stress
Gs	Stomatal Conductance
ICP	Inductively Coupled Plasma
NDVI	Normalized Difference Vegetation Index
pH	Potential of Hydrogen
PSII	Photosystem II
TA.XTPlus	Texture Analyzer
TACy	Total Anthocyanin Content
WUE	Water Use efficiency
Y(II)	Effective Quantum Yield of PSII
