# Supplementary material for: The Impact of Elevated Soil pH Levels on Cranberry Growth, Physiology, and Metabolites
Source: Plants (Basel). 2025 Sep 11;14(18):2833. doi: 10.3390/plants14182833 (PMC12473267; doi:10.3390/plants14182833)
Supplement: Supplementary file 1 [file plants-14-02833-s001.zip › plants-3811100-supplementary.pdf]

1. Supplementary table and figures

The impact of elevated soil pH levels on cranberry growth, physiology, and metabolites

Mura Jyostna Devi[1,2*], Jinyoung Barnaby[3], Jessica Rohde[1,2], Yi Wang[2], Lorraine Rodriguez-Bonilla[2], Juan Zalapa[1,2], Amaya Atucha[2], Giverson Mupambi[4]

| Water Nutrients | Control | High pH1 | High pH2 | High pH3 | High pH4 | Significance Level |
|---|---|---|---|---|---|---|
| Alkalinity as CaCO3 (mg/L) | 10 ± 0.5 | 107 ± 1.5 | 107 ± 1.0 | 112 ± 4.0 | 130 ± 2.5 | **** |
| Boron (mg/L) | 0.23 ± 0.01 | 0.03 ± 0.00 | 0.03 ± 0.00 | 0.14 ± 0.00 | 0.14 ± 0.00 | * |
| Total Calcium (mg/L) | 3.3 ± 0.20 | 2.0 ± 0.10 | 23 ± 3.0 | 82 ± 4.0 | 95 ± 3.0 | *** |
| Chloride (mg/L) | 0.8 ± 0.07 | 6.0 ± 0.50 | 4.0 ± 0.50 | 19 ± 0.50 | 22 ± 0.20 | *** |
| Conductivity (umhos/cm) | 25 ± 1.00 | 225 ± 1.00 | 266 ± 3.00 | 430 ± 5.00 | 488 ± 4.00 | **** |
| Total Iron (mg/L) | 0.24 ± 0.005 | 0.80 ± 0.005 | 0.80 ± 0.005 | 0.05 ± 0.00 | 0.20 ± 0.01 | * |
| Total Hardness (mg/L) | 12 ± 1.00 | 133 ± 1.00 | 133 ± 1.50 | 309 ± 2.00 | 341 ± 10.0 | **** |
| Total Potassium (mg/L) | 1.0 ± 0.10 | 6.21 ± 0.13 | 5.79 ± 0.12 | 4.0 ± 0.10 | 2.78 ± 0.14 | ** |
| Total Magnesium (mg/L) | 1.10 ± 0.10 | 10.3 ± 0.10 | 12.1 ± 0.10 | 25 ± 0.10 | 25 ± 0.10 | ** |
| Total Manganese (mg/L) | 0.05 ± 0.01 | 0.04 ± 0.01 | 0.03 ± 0.00 | 0.02 ± 0.00 | 0.02 ± 0.01 | NS |
| Total Sodium (mg/L) | 1.8 ± 0.2 | 2.1 ± 0.15 | 1.7 ± 0.10 | 3.05 ± 0.15 | 3.52 ± 0.12 | * |
| Nitrate as N (mg/L) | 0.1 ± 0.05 | ND | 1.4 ± 0.15 | 9.04 ± 1.5 | 11 ± 1.7 | *** |
| Orthophosphorus (mg/L) | 0.04 ± 0.01 | 0.04 ± 0.00 | 0.04 ± 0.00 | 0.04 ± 0.00 | 0.02 ± 0.00 | NS |
| Sulfate (mg/L) | 0.10 ± 0.00 | 2.0 ± 0.02 | 8.0 ± 0.05 | 49 ± 0.10 | 50 ± 2.00 | *** |

Table S1: Average + S.E. of water components of the irrigation water received by control and four high pH cranberry beds in year 3 during spring (after bud break -initial growing stages) and fall (mature fruit stage). The significance levels were calculated based on ANOVA Tukey-Kramer model, NS – Nonsignificant, * P<0.05, ** P<0.01, *** P<0.001, and **** P<0.0001.

| Spring | Control | High pH1 | High pH2 | High pH3 | High pH4 | Significance Level |
|---|---|---|---|---|---|---|
| C (%) | 0.5 ± 0.1 | 0.2 ± 0.0 | 0.1 ± 0.0 | 0.2 ± 0.1 | 0.3 ± 0.0 | ** |
| N (%) | 0.008 ± 0.001 | 0.01 ± 0.001 | 0.01 ± 0.001 | 0.02 ± 0.001 | 0.03 ± 0.001 | *** |
| P (mg/kg) | 51.4 ± 13.3 | 18.5 ± 1.7 | 95.2 ± 10.4 | 22.3 ± 10.5 | 101.5 ± 13.4 | ** |
| K (mg/kg) | 13.7 ± 0.6 | 14.0 ± 1.0 | 25.7 ± 4.2 | 19.3 ± 4.0 | 36.33 ± 14.4 | NS |
| Ca (mg/kg) | 53.7 ± 15.3 | 240.0 ± 11.1 | 470.7 ± 96.4 | 213.3 ± 73.7 | 485.0 ± 67.5 | *** |
| Mg (mg/kg) | 15.7 | 90.3 ± 7.6 | 81.0 ± 16.8 | 37.3 ± 3.52 | 94.3 ± 9.0 | *** |
| S (mg/kg) | 6.2 ± 1.3 | 8.3 ± 1.1 | 11.3 ± 1.9 | 17.5 ± 18.6 | 14.7 ± 1.3 | NS |
| Na (mg/kg) | 5.7 ± 0.4 | 5.1 ± 0.7 | 4.9 ± 0.1 | 3.8 ± 0.1 | 5.4 ± 0.2 | *** |
| Fe (mg/kg) | 98.7 ± 16.3 | 117.0 ± 3.0 | 122.7 ± 7.6 | 83.3 ± 19.4 | 148.0 ± 16.1 | * |
| Mn (mg/kg) | 3.0 ± 0.3 | 5.4 ± 0.4 | 10.4 ± 2.7 | 4.2 ± 1.7 | 6.6 ± 1.4 | ** |
| Zn (mg/kg) | 0.5 ± 0.0 | 1.1 ± 0.4 | 2.8 ± 0.7 | 0.8 ± 0.4 | 3.3 ± 1.3 | ** |
| Cu (mg/kg) | 0.4 ± 0.1 | 1.0 ± 0.4 | 2.8 ± 0.9 | 0.4 ± 0.2 | 3.1 ± 0.3 | *** |
| B (mg/kg) | 0.5 ± 0.0 | 0.5 ± 0.0 | 0.6 ± 0.0 | 0.4 ± 0.1 | 0.6 ± 0.1 | *** |
| **Fall** | | | | | | |
| C (%) | 1.4 ± 0.03 | 0.6 ± 0.11 | 0.6 ± 011 | 0.8 ± 0.20 | 0.81 ± 0.11 | ** |
| N (%) | 0.05 ± 0.001 | 0.03 ± 0.003 | 0.02 ± 0.003 | 0.03 ± 0.007 | 0.03 ± 0.002 | NS |
| P (mg/kg) | 33.4 ± 1.45 | 18.5 ± 0.95 | 46.8 ± 8.0 | 60.7 ± 13.0 | 57.2 ± 6.14 | ** |
| K (mg/kg) | 23.5 ± 1.5 | 14.0 ± 0.57 | 21.3 ± 2.1 | 36.0 ± 2.3 | 26.3 ± 3.17 | NS |
| Ca (mg/kg) | 345.0 ± 24 | 240.0 ± 6.4 | 370.6 ± 65 | 571.0 ± 101 | 364.6 ± 10.4 | *** |
| Mg (mg/kg) | 85.0 ± 4.0 | 90.3 ± 4.4 | 86.6 ± 9.0 | 106.3 ± 18.2 | 72.3 ± 4.4 | *** |
| S (mg/kg) | 21.4 ± 0.3 | 8.3 ± 0.6 | 16.7 ± 4.3 | 28.6 ± 1.7 | 24.9 ± 3.8 | ** |
| Na (mg/kg) | 4.7 ± 0.5 | 5.1 ± 0.3 | 5.9 ± 0.6 | 4.9 ± 0.05 | 4.1 ± 0.4 | NS |
| Fe (mg/kg) | 84.0 ± 4 | 117.0 ± 1.7 | 97.0 ± 12.0 | 114.0 ± 11.2 | 107.0 ± 3.0 | NS |
| Mn (mg/kg) | 3.4 ± 0.2 | 5.4 ± 0.3 | 4.2 ± 0.6 | 6.9 ± 2.4 | 4.5 ± 0.29 | NS |
| Zn (mg/kg) | 3.5 ± 0.0 | 1.1 ± 0.3 | 3.2 ± 0.3 | 3.1 ± 0.7 | 2.9 ± 0.4 | * |
| Cu (mg/kg) | 2.1 ± 0.2 | 1.0 ± 0.3 | 1.8 ± 0.2 | 2.1 ± 0.5 | 2.1 ± 0.15 | NS |
| B (mg/kg) | 0.5 ± 0.0 | 0.5 ± 0.0 | 0.5 ± 0.0 | 0.6 ± 0.03 | 0.6 ± 0.03 | ** |

Table S2: Macro and micronutrients from soils collected from different soil pH cranberry beds in year 3 during spring (after bud break -initial growing stages) and fall (mature fruit stage). The significance levels were calculated based on ANOVA Tukey-Kramer model, NS – Nonsignificant, * P<0.05, ** P<0.01, *** P<0.001, and **** P<0.0001.

| Year 1 | Control | ±S.E. | High pH1 | ±S.E. | High pH2 | ±S.E. | Significance Level |
|---|---|---|---|---|---|---|---|
| **Upright Stem** | | | | | | | |
| Na (ppm) | 111.6 | 9.0 | 33.8 | 2.8 | 25.2 | 1.3 | *** |
| Fe (ppm) | 949.4 | 135.2 | 647.6 | 74.3 | 451.0 | 71.2 | ** |
| Mn (ppm) | 619.0 | 65.7 | 186.9 | 5.1 | 138.4 | 12.3 | *** |
| Zn (ppm) | 22.7 | 1.5 | 15.1 | 0.8 | 13.5 | 0.3 | * |
| Cu (ppm) | 7.5 | 0.3 | 5.2 | 0.2 | 5.9 | 0.1 | * |
| B (ppm | 9.7 | 0.4 | 10.0 | 1.2 | 6.8 | 0.2 | NS |
| **Upright Leaves** | | | | | | | |
| Na (ppm) | 59.8 | 3.5 | 20.4 | 2.5 | 15.2 | 1.0 | *** |
| Fe (ppm) | 687.6 | 69.9 | 607.8 | 107.1 | 509.6 | 48.5 | ** |
| Mn (ppm) | 404.4 | 44.9 | 133.8 | 8.6 | 107.3 | 8.8 | *** |
| Zn (ppm) | 25.9 | 0.7 | 16.9 | 0.9 | 17.1 | 0.7 | NS |
| Cu (ppm) | 3.8 | 0.1 | 2.4 | 0.3 | 2.4 | 0.1 | NS |
| B (ppm | 41.1 | 2.3 | 27.0 | 1.7 | 23.4 | 1.3 | NS |
| **Small Fruit Stage** | | | | | | | |
| Na (ppm) | 47.8 | 4.4 | 20.6 | 0.9 | 18.0 | 0.9 | *** |
| Fe (ppm) | 21.0 | 2.9 | 12.6 | 0.7 | 12.6 | 0.7 | *** |
| Mn (ppm) | 25.0 | 2.8 | 4.4 | 0.3 | 2.5 | 0.5 | *** |
| Zn (ppm) | 9.5 | 0.2 | 8.1 | 0.2 | 8.3 | 0.2 | NS |
| Cu (ppm) | 4.3 | 0.3 | 4.4 | 0.1 | 4.9 | 0.4 | NS |
| B (ppm | 6.8 | 0.2 | 6.4 | 0.2 | 6.0 | 0.4 | NS |
| **Medium Fruit Stage** | | | | | | | |
| Na (ppm) | 46.4 | 3.1 | 18.3 | 0.5 | 19.0 | 0.9 | *** |
| Fe (ppm) | 15.3 | 0.8 | 14.5 | 2.0 | 17.8 | 1.9 | NS |
| Mn (ppm) | 21.5 | 2.5 | 3.7 | 0.8 | 1.7 | 0.4 | *** |
| Zn (ppm) | 12.0 | 4.0 | 7.1 | 0.1 | 7.2 | 0.1 | NS |
| Cu (ppm) | 4.6 | 0.7 | 3.6 | 0.2 | 3.4 | 0.0 | NS |
| B (ppm | 6.5 | 0.3 | 6.0 | 0.5 | 5.4 | 0.3 | NS |
| **Mature Fruit Stage** | | | | | | | |
| Na (ppm) | 39.2 | 1.8 | 16.3 | 0.5 | 13.0 | 0.9 | **** |
| Fe (ppm) | 20.2 | 1.3 | 13.3 | 1.3 | 13.8 | 0.3 | **** |
| Mn (ppm) | 17.8 | 1.8 | 2.3 | 0.6 | 1.3 | 0.7 | **** |
| Zn (ppm) | 6.8 | 0.1 | 6.2 | 0.1 | 6.1 | 0.1 | NS |
| Cu (ppm) | 4.2 | 0.3 | 3.3 | 0.1 | 3.2 | 0.0 | NS |
| B (ppm | 5.6 | 0.2 | 5.1 | 0.4 | 4.8 | 0.2 | NS |

Table S3: Average ± S.E. of different micronutrients sodium (Na), iron (Fe), manganese (Mn), zinc (Zn), copper (Cu), and boron (B) in leaves and stems collected in September (at harvest) and fruits at different maturity stages of control and two high soil pH cranberry beds in year 1. The significance levels were calculated based on ANOVA Tukey-Kramer model, NS – Nonsignificant, * P<0.05, ** P<0.01, *** P<0.001, and **** P<0.0001.

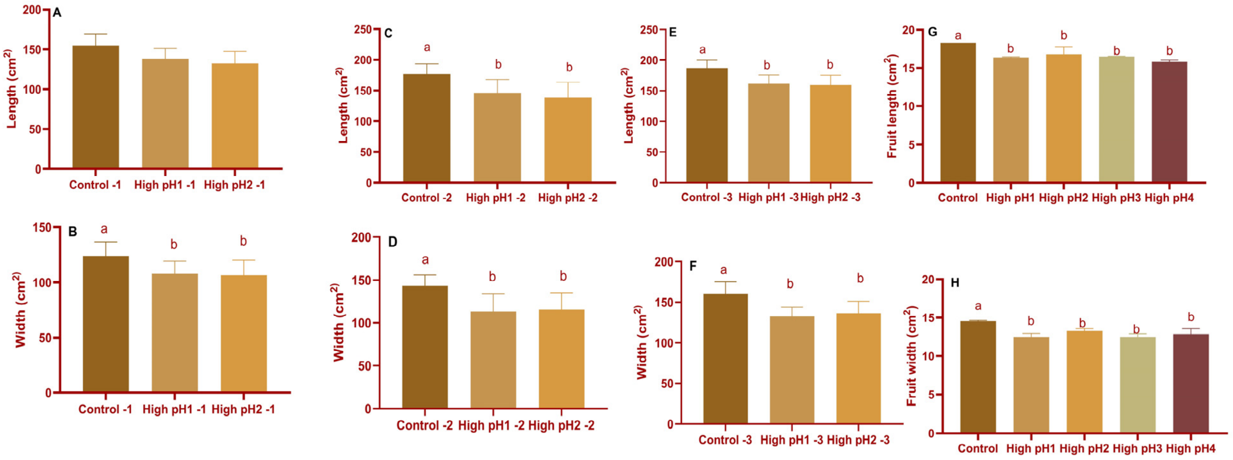


Figure S1: The mean ± standard error (SE) of fruit length and width of fruits grown under both control and high soil pH conditions. The figures A and B are length and width measured during small fruit stage, C and D during medium fruit and E and F during mature stage in the first year of the experiment. The figures G and H are fruit and length measured in year 3 in four high pH beds and one control bed. The bars represented by different alphabet were significantly different (P<0.05) from each other based on Tukey's-Kramer.

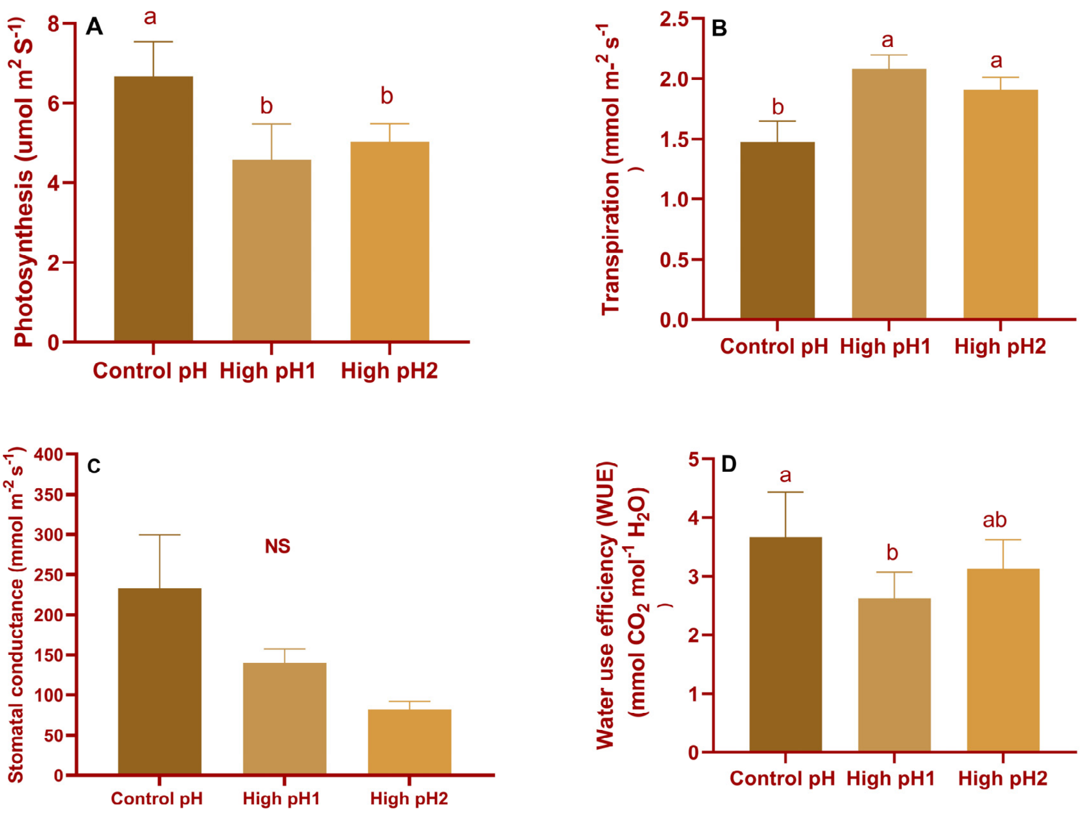


Figure S2: Mean ± SE of photosynthesis, transpiration, stomatal conductance, and water use efficiency of cranberry uprights measured during first week of June 2022 (year 3) in old upright shoots/early developmental stages. The bars represented by different alphabet are significantly different (P<0.05) from each other based on ANOVA - Tukey's-Kramer.

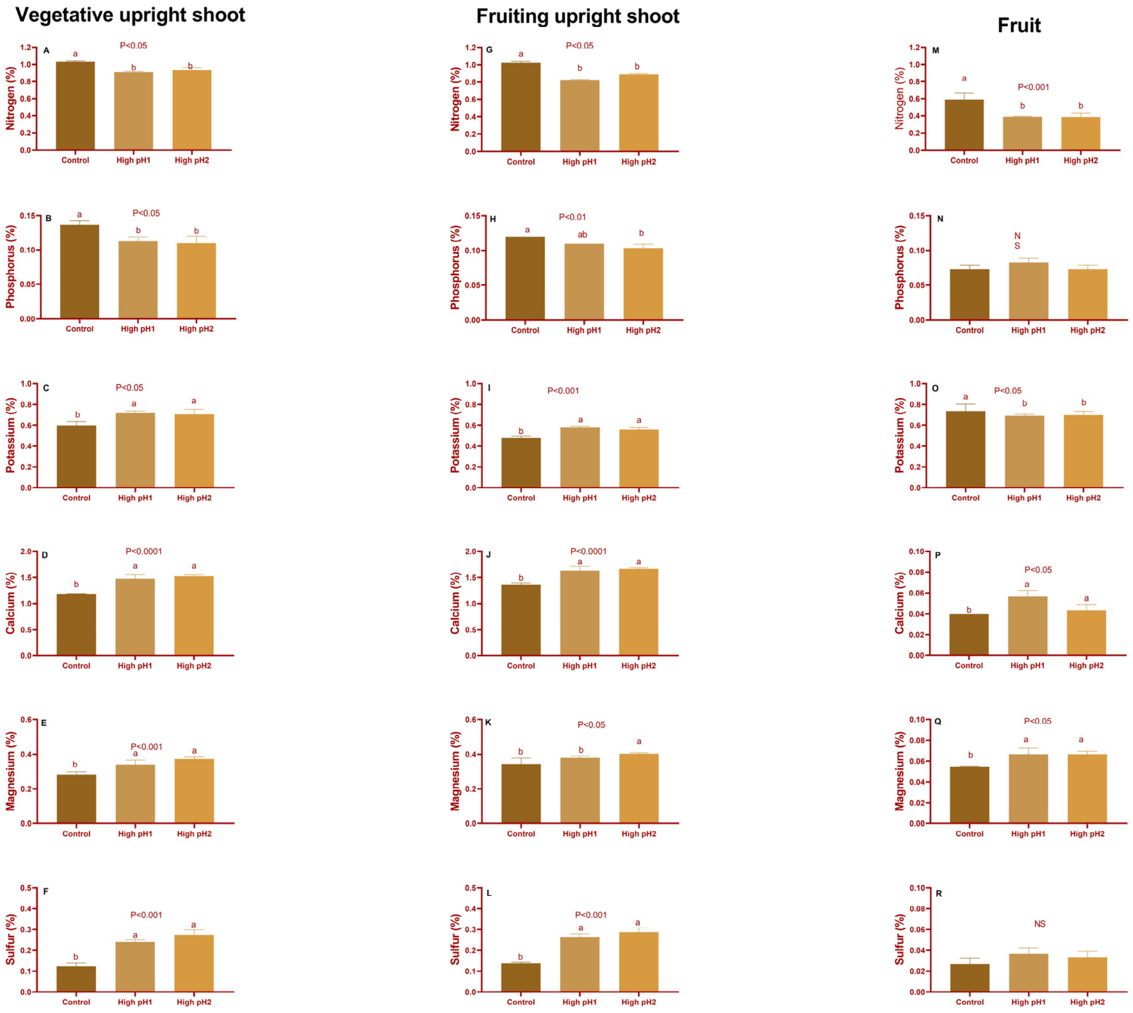


Figure S3: Mean ± SE of macro nutrients nitrogen (A,G, and M), phosphorus (B, H, and N), potassium (C, I, and O), calcium (D, J, and P), magnesium (E, K, and Q) and sulfur (F, L, and R) of cranberry vegetative and fruiting uprights in year 2. The bars represented by different alphabet are significantly different (P<0.05) from each other based on Tukey's-Kramer. NS - Nonsignificant

| Year 2 | Control | ±S.E. | High pH1 | ±S.E. | High pH2 | ±S.E. | Significance Level |
|---|---|---|---|---|---|---|---|
| **Vegetative Upright Shoot** | | | | | | | |
| Na (ppm) | 18.2 | 4.1 | 7.5 | 0.2 | 9.7 | 1.9 | NS |
| Fe (ppm) | 236.0 | 21.1 | 269.1 | 33.1 | 277.9 | 26.4 | NS |
| Mn (ppm) | 148.7 | 21.3 | 166.7 | 4.2 | 168.0 | 9.9 | NS |
| Zn (ppm) | 20.7 | 1.2 | 19.6 | 0.8 | 21.8 | 1.4 | NS |
| Cu (ppm) | 2.9 | 0.1 | 1.9 | 0.0 | 2.1 | 0.0 | *** |
| B (ppm | 54.4 | 1.4 | 47.7 | 1.8 | 40.9 | 1.8 | ** |
| **Fruiting Upright Shoot** | | | | | | | |
| Na (ppm) | 24.9 | 3.7 | 8.9 | 1.5 | 7.4 | 0.5 | ** |
| Fe (ppm) | 272.9 | 39.4 | 328.5 | 56.3 | 273.3 | 20.4 | NS |
| Mn (ppm) | 211.2 | 19.1 | 175.0 | 3.5 | 198.1 | 13.1 | NS |
| Zn (ppm) | 22.8 | 0.9 | 18.1 | 1.3 | 19.4 | 1.5 | NS |
| Cu (ppm) | 3.0 | 0.1 | 1.8 | 0.1 | 1.8 | 0.0 | *** |
| B (ppm | 55.4 | 6.4 | 43.8 | 3.2 | 36.4 | 0.9 | * |
| **Fruit** | | | | | | | |
| Na (ppm) | 46.8 | 1.3 | 15.1 | 0.4 | 15.5 | 1.0 | *** |
| Fe (ppm) | 15.8 | 1.8 | 18.8 | 1.0 | 19.5 | 3.2 | NS |
| Mn (ppm) | 3.8 | 1.0 | 3.3 | 0.7 | 2.6 | 0.5 | NS |
| Zn (ppm) | 5.5 | 0.2 | 6.3 | 0.3 | 6.7 | 0.4 | NS |
| Cu (ppm) | 3.6 | 0.4 | 3.2 | 0.1 | 3.1 | 0.3 | NS |
| B (ppm | 3.8 | 0.1 | 4.9 | 0.0 | 4.3 | 0.3 | * |

Table S4: The mean ± standard error (SE) of micronutrients sodium (Na), iron (Fe), manganese (Mn), zinc (Zn), copper (Cu), and boron (B) in cranberry vegetative, fruiting uprights, and fruits collected during harvest/mature fruit stage in year 2. Based on Tukey-Kramer model, the significance levels are NS – Nonsignificant, * P<0.05, ** P<0.01, *** P<0.001, and **** P<0.0001.

| Year 3 | Control | ±S.E. | High pH1 | ±S.E. | High pH2 | ±S.E. | High pH3 | ±S.E. | High pH4 | ±S.E. | Significance Level |
|---|---|---|---|---|---|---|---|---|---|---|---|
| **Upright leaves (Spring)** | | | | | | | | | | | |
| Na (ppm) | 139.4 | 26.8 | 32.3 | 9.8 | 42.4 | 2.9 | 40.4 | 3.0 | 45.9 | 5.7 | ** |
| Fe (ppm) | 1647.7 | 262.8 | 84.7 | 13.2 | 116.7 | 17.8 | 131.7 | 14.7 | 76.5 | 6.5 | **** |
| Mn (ppm) | 217.2 | 33.0 | 50.5 | 16.3 | 172.1 | 37.3 | 75.9 | 4.2 | 78.6 | 10.8 | ** |
| Zn (ppm) | 22.6 | 0.1 | 18.6 | 1.5 | 25.1 | 2.3 | 16.9 | 1.1 | 22.8 | 1.3 | * |
| Cu (ppm) | 3.2 | 0.1 | 4.2 | 0.2 | 2.9 | 0.2 | 2.5 | 0.3 | 4.5 | 0.3 | *** |
| B (ppm | 45.3 | 3.9 | 27.2 | 1.3 | 34.7 | 1.1 | 29.2 | 1.2 | 23.9 | 1.5 | *** |
| **Upright leaves (Fall)** | | | | | | | | | | | |
| Na (ppm) | 28.5 | 10.5 | 10.0 | 1.0 | 6.4 | 0.2 | 11.6 | 5.7 | 17.2 | 4.4 | NS |
| Fe (ppm) | 594.3 | 95.2 | 327.3 | 15.3 | 275.0 | 95.3 | 457.3 | 132.5 | 311.7 | 113.3 | NS |
| Mn (ppm) | 187.3 | 47.8 | 211.9 | 17.1 | 59.4 | 9.0 | 117.6 | 27.9 | 100.8 | 14.9 | * |
| Zn (ppm) | 19.7 | 1.5 | 16.7 | 0.9 | 12.7 | 0.4 | 15.8 | 1.3 | 19.3 | 0.8 | ** |
| Cu (ppm) | 2.8 | 0.0 | 1.8 | 0.1 | 2.2 | 0.1 | 2.2 | 0.4 | 3.4 | 0.7 | NS |
| B (ppm | 44.9 | 1.5 | 37.2 | 3.8 | 40.2 | 3.1 | 35.5 | 6.2 | 25.7 | 2.0 | * |
| **Upright Stem (Spring)** | | | | | | | | | | | |
| Na (ppm) | 74.3 | 6.3 | 48.4 | 1.3 | 48.2 | 7.2 | 47.1 | 4.8 | 49.2 | 5.4 | * |
| Fe (ppm) | 893.0 | 183.5 | 94.3 | 4.1 | 69.3 | 4.8 | 113.3 | 27.5 | 118.0 | 31.0 | *** |
| Mn (ppm) | 405.7 | 96.7 | 77.7 | 28.3 | 346.7 | 92.4 | 97.4 | 9.9 | 107.7 | 1.3 | * |
| Zn (ppm) | 17.8 | 1.4 | 10.3 | 0.6 | 23.6 | 1.3 | 15.5 | 0.3 | 13.1 | 1.0 | **** |
| Cu (ppm) | 7.1 | 1.2 | 5.3 | 0.3 | 4.8 | 0.2 | 4.3 | 0.2 | 5.7 | 0.3 | NS |
| B (ppm | 6.9 | 0.4 | 6.8 | 0.5 | 8.1 | 0.6 | 7.4 | 0.2 | 6.0 | 0.2 | NS |
| **Upright Stem (Fall)** | | | | | | | | | | | |
| (Na (ppm) | 55.8 | 12.7 | 25.4 | 1.7 | 35.8 | 11.0 | 38.9 | 12.1 | 62.5 | 7.3 | NS |
| Fe (ppm) | 415.0 | 120.9 | 276.3 | 74.6 | 75.7 | 29.7 | 207.0 | 87.8 | 404.3 | 195.2 | NS |
| Mn (ppm) | 274.9 | 76.6 | 273.7 | 33.5 | 80.1 | 19.5 | 138.7 | 27.4 | 122.0 | 23.6 | * |
| Zn (ppm) | 16.0 | 1.0 | 15.2 | 0.9 | 11.4 | 0.9 | 17.6 | 1.2 | 12.9 | 1.1 | * |
| Cu (ppm) | 6.5 | 0.3 | 4.6 | 0.2 | 5.2 | 0.0 | 4.4 | 0.6 | 5.8 | 0.6 | * |
| B (ppm | 6.0 | 0.2 | 7.4 | 0.7 | 6.2 | 0.2 | 6.1 | 0.4 | 5.5 | 0.4 | NS |
| **Fruit (Fall)** | | | | | | | | | | | |
| Na (ppm) | 45.8 | 0.0 | 25.4 | 3.6 | 15.5 | 1.0 | 33.3 | 5.9 | 35.0 | 3.4 | * |
| Fe (ppm) | 217.0 | 0.0 | 189.0 | 78.8 | 19.5 | 3.2 | 167.3 | 73.1 | 92.7 | 56.7 | NS |
| Mn (ppm) | 9.2 | 0.0 | 5.2 | 2.2 | 2.6 | 0.5 | 3.1 | 1.4 | 1.6 | 0.1 | NS |
| Zn (ppm) | 5.2 | 0.0 | 6.6 | 0.9 | 6.7 | 0.4 | 5.9 | 0.8 | 7.0 | 0.3 | NS |
| Cu (ppm) | 2.9 | 0.0 | 2.5 | 0.6 | 3.1 | 0.3 | 2.4 | 0.3 | 3.4 | 0.1 | NS |
| B (ppm | 4.8 | 0.0 | 4.2 | 0.4 | 4.3 | 0.3 | 3.4 | 0.5 | 2.9 | 0.2 | NS |

Table S5: The mean ± standard error (SE) of micronutrients sodium (Na), iron (Fe), manganese (Mn), zinc (Zn), copper (Cu), and boron (B) in cranberry upright leaves and stem collected in fall and spring and fruits collected during harvest/mature fruit stage in year 3. According to Tukey-Kramer ANOVA analysis, the significance levels represented here are NS – Nonsignificant, * P<0.05, ** P<0.01, *** P<0.001, and **** P<0.0001.

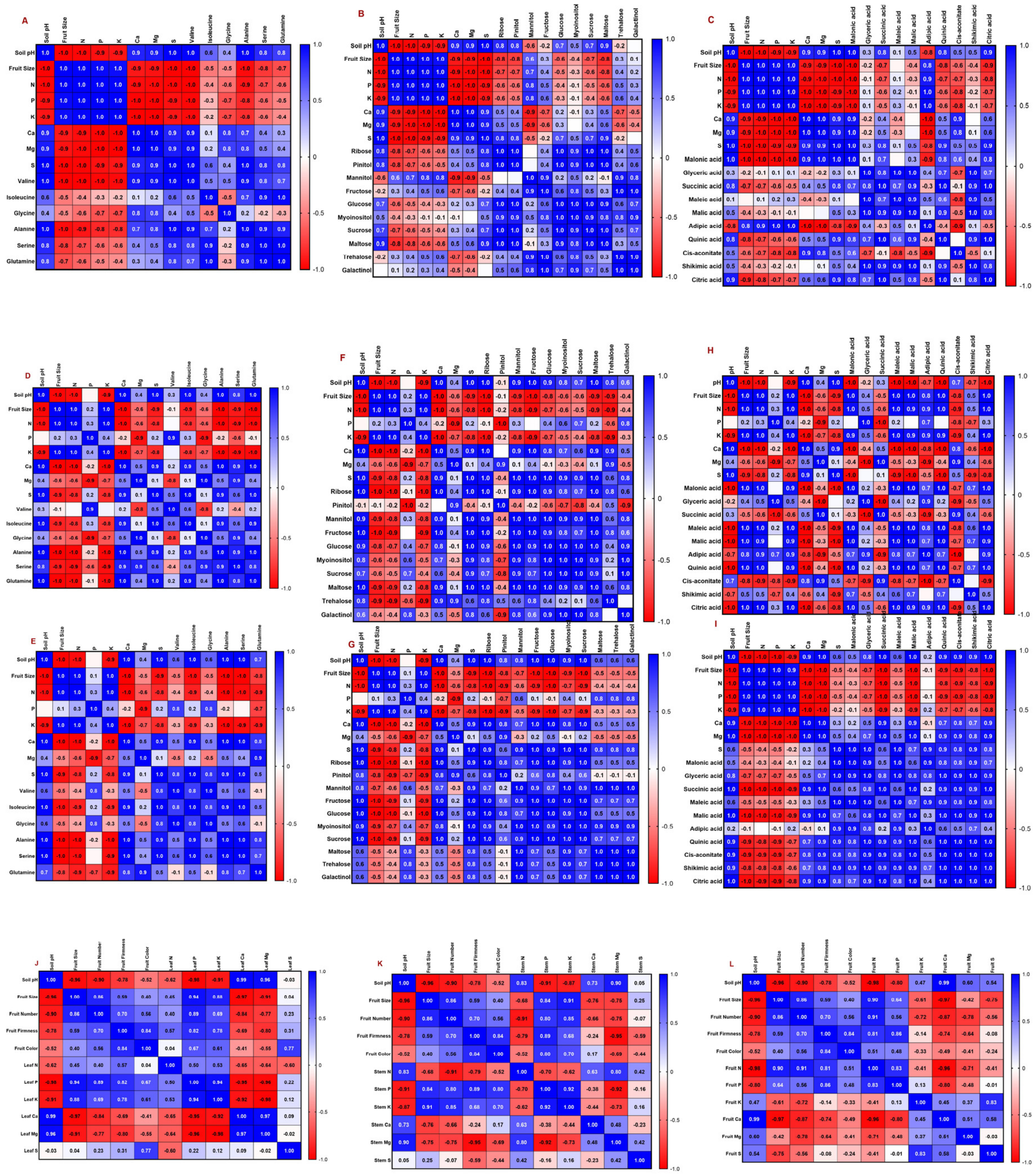

Figure S4 : Correlation matrix of soil pH, fruit traits, nutrients and metabolites of cranberry shoots and fruits in year 1 and 3

Correlation matrix of soil pH, fruit size, and macro nutrients with A) Amino Acids B) Carbohydrates, and C)Organic Acids of the samples collected in September year 1; fruit size, fruit nutrients, and fruit metabolites relation with D) Amino Acids E) Carbohydrates and F) Organic Acids of the samples collected in August and year 1; soil pH, fruit size, fruit nutrients, and fruit metabolites relation with G) Amino Acids H) Carbohydrates and I) Organic Acids of the samples collected in September in year 1; Correlation

matrix of soil pH, fruit size, fruit firmness, and fruit nutrients with J) Leaf nutrients K) Shoot nutrients, and L)Fruit nutrients of the samples collected in October, year 3
The cell value indicates Pearson' correlation coefficient value. The cells represented with red are negatively related parameters and the blue ones are positively related. The Pearson values above 0.86 were significant at P<0.05.

| | Year 1 - Medium Fruit | | Year 1 - Mature Fruit | | Year 3 - Mature Fruit | | | | |
|---|---|---|---|---|---|---|---|---|---|
| | Soil pH | Fruit Size | Soil pH | Fruit Size | Soil pH | Fruit Size | Fruit Number | Fruit Firmness | Fruit Color |
| Soil pH | - | **0.05** | - | **0.03** | - | **0.01** | **0.02** | 0.06 | 0.18 |
| Fruit Size | **0.05** | | **0.03** | - | **0.01** | | **0.03** | 0.15 | 0.25 |
| Fruit Number | - | - | - | - | **0.02** | **0.03** | | 0.10 | 0.16 |
| Fruit Firmness | - | - | - | - | 0.06 | 0.15 | 0.10 | | **0.04** |
| Fruit Color | - | - | - | - | 0.18 | 0.25 | 0.16 | **0.04** | |
| N | 0.09 | **0.03** | 0.06 | **0.03** | **0.00** | **0.02** | **0.02** | **0.05** | 0.19 |
| P | 0.49 | 0.42 | **0.04** | **0.01** | **0.05** | 0.12 | 0.16 | **0.03** | 0.21 |
| K | 0.11 | **0.04** | 0.13 | 0.10 | 0.21 | 0.14 | 0.08 | 0.41 | 0.29 |
| Ca | **0.04** | **0.02** | 0.11 | **0.05** | **0.00** | **0.00** | **0.03** | 0.08 | 0.20 |
| Mg | 0.38 | 0.31 | **0.04** | **0.01** | 0.14 | 0.24 | 0.06 | 0.12 | 0.25 |
| S | 0.08 | 0.15 | 0.29 | 0.32 | 0.18 | 0.07 | 0.16 | 0.45 | 0.35 |
| Malonic acid | **0.01** | 0.07 | 0.33 | 0.36 | - | - | - | - | - |
| Glyceric acid | 0.43 | 0.36 | 0.21 | 0.24 | - | - | - | - | - |
| Succinic acid | 0.39 | 0.33 | **0.01** | **0.02** | - | - | - | - | - |
| Maleic acid | **0.04** | **0.03** | 0.29 | 0.32 | - | - | - | - | - |
| Malic acid | **0.01** | 0.08 | **0.03** | 0.06 | - | - | - | - | - |
| Adipic acid | 0.25 | 0.19 | 0.44 | 0.47 | - | - | - | - | - |
| Quinic acid | **0.00** | 0.07 | 0.13 | 0.16 | - | - | - | - | - |
| Cis-aconitate | 0.26 | 0.19 | 0.10 | 0.13 | - | - | - | - | - |
| Shikimic acid | 0.26 | 0.32 | 0.16 | 0.19 | - | - | - | - | - |
| Citric acid | 0.09 | **0.03** | 0.06 | 0.09 | - | - | - | - | - |
| Valine | 0.41 | 0.48 | 0.29 | 0.32 | - | - | - | - | - |
| Isoleucine | 0.10 | 0.16 | 0.07 | 0.10 | - | - | - | - | - |
| Glycine | 0.38 | 0.31 | 0.29 | 0.32 | - | - | - | - | - |
| Alanine | **0.04** | **0.02** | **0.04** | **0.01** | - | - | - | - | - |
| Serine | 0.21 | 0.14 | **0.00** | **0.03** | - | - | - | - | - |
| Glutamine | **0.02** | **0.05** | 0.25 | 0.22 | - | - | - | - | - |
| Ribose | **0.01** | 0.06 | **0.03** | **0.00** | - | - | - | - | - |
| Pinitol | 0.46 | 0.48 | 0.23 | 0.20 | - | - | - | - | - |
| Mannitol | 0.10 | 0.17 | 0.22 | 0.25 | - | - | - | - | - |
| Fructose | **0.01** | 0.08 | **0.05** | 0.08 | - | - | - | - | - |
| Glucose | 0.15 | 0.22 | **0.03** | **0.00** | - | - | - | - | - |
| Myoinositol | 0.22 | 0.28 | 0.15 | 0.17 | - | - | - | - | - |
| Sucrose | 0.25 | 0.31 | **0.05** | 0.08 | - | - | - | - | - |
| Maltose | 0.08 | 0.15 | 0.29 | 0.32 | - | - | - | - | - |
| Trehalose | 0.21 | 0.14 | 0.29 | 0.32 | - | - | - | - | - |
| Galactinol | 0.29 | 0.35 | 0.29 | 0.32 | - | - | - | - | - |

Table S6 : Correlation matrix of soil pH, fruit traits, fruit nutrients, and fruit metabolites of cranberry fruits in years 1 and 3.
Year 1 fruit data includes different metabolites and nutrients, however, year 3 had only nutrients. The cell indicates the P value of each correlation between parameters. Significantly correlations are marked in bold and red.

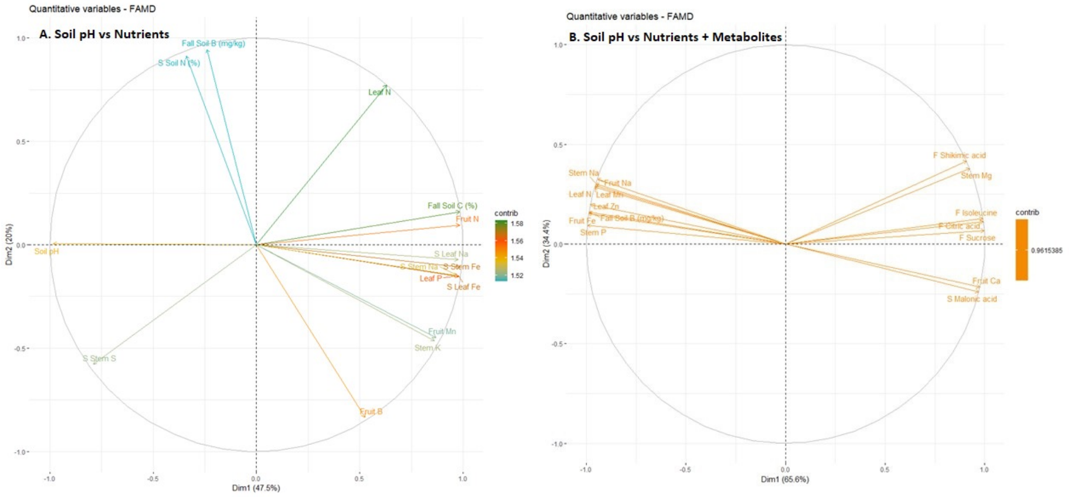


Figure S5: Quantitative variables of the first two dimensions resulting from factor analysis of mixed data (FAMD) of A) Soil pH Vs nutrients B) Soil pH vs Nutrients (soil, plant, and fruit) and Metabolites (Shoot and Fruit). Plot shows the top 15 contributing factors. In figure B and C, "S" in front of nutrient name represents Spring. In figure D, S and F in front of metabolite name represents, shoot and fall, respectively. The data set D includes data from 2020 (year 1) since the metabolites were measured only in that experimental year.
